# Host Genetic and Environmental Factors Shape the Composition and Function of Gut Microbiota in Populations Living at High Altitude

**DOI:** 10.1155/2020/1482109

**Published:** 2020-02-28

**Authors:** Kang Li, Wei Peng, Youlian Zhou, Yi Ren, Jianhua Zhao, Xiangsheng Fu, Yuqiang Nie

**Affiliations:** ^1^High Altitude Medical Research Institute, People's Hospital of Tibet Autonomous Region, Lhasa 850000, China; ^2^Department of Gastroenterology, Guangzhou First People's Hospital of Guangzhou Medical University, Guangzhou 510180, China; ^3^Department of Gastroenterology, The Affiliated Hospital of Southwest Medical University, Luzhou 646000, China; ^4^Shanghai Majorbio Bio-Pharm Technology Co., Ltd., Shanghai 200000, China; ^5^Department of Gastroenterology, The Affiliated Hospital of North Sichuan Medical College, Nanchong 637000, China

## Abstract

The human gut microbiota is affected by genetic and environmental factors. It remains unclear how host genetic and environmental factors affect the composition and function of gut microbiota in populations living at high altitudes. We used a metagenome-wide analysis to investigate the gut microbiota composition in 15 native Tibetans and 12 Hans living on the Tibetan Plateau. The composition of gut microbiota differed significantly between these two groups (*P* < 0.05). The *Planctomycetes* was the most abundant phyla both in native Tibetans and in Hans. Furthermore, the most relatively abundant phyla for native Tibetans were *Bacteroidetes* (15.66%), *Firmicutes* (11.10%), *Proteobacteria* (1.32%), *Actinobacteria* (1.10%), and *Tenericutes* (0.35%), while the most relatively abundant phyla for Hans were *Bacteroidetes* (16.28%), *Firmicutes* (8.41%), *Proteobacteria* (2.93%), *Actinobacteria* (0.49%), and *Cyanobacteria* (0.21%). The abundance of the majority of genera was significantly higher in Tibetans than in Hans (*P* < 0.01). The number of microbial genes was 4.9 times higher in Tibetans than in Hans. The metabolic pathways and clusters of orthologous groups differed significantly between the two populations (*P* < 0.05). The abundance of carbohydrate-active enzyme modules and antibiotic resistance genes was significantly lower in Tibetans compared to Hans (*P* < 0.05). Our results suggest that different genetic factors (race) and environmental factors (diets and consumption of antibiotics) may play important roles in shaping the composition and function of gut microbiota in populations living at high altitudes.

## 1. Introduction

The gut microbiota plays important roles in maintaining normal physiological and metabolic functions of the human body [1, 2]. It contains at least 100 times as many genes as the human body itself [3]. These microbes help aid food digestion and other metabolic capabilities, produce certain vitamins, and protect against infections from other microorganisms. The gut microbiota also plays an important role in the immune system [4].

About one-third of the gut microbiota is common to most people [5], but the rest differs among different genetic backgrounds and people with different environmental factors [6]. The composition of the gut microbiota may be unique to each individual, varying based on age, diet, and health status [7, 8]. A recent study showed that diet could rapidly change the composition of the gut microbiome [9]. Several studies have also shown significant differences of the gut microbiome between people of nonindustrialized societies and those with westernized lifestyles [10, 11].

Another factor that affects the gut microbiota is geographic location and its associated natural environment [8, 12]. Native Tibetans who feed on meat and dairy products live on the Tibetan Plateau, the world's highest and largest plateau, with an average elevation exceeding 4,500 m. A recent study showed that the number of bacterial species in the gut microbiota of Hans was dramatically decreased several years after migration from a low to high altitude [13].

The native Tibetans have developed unique lifestyles and dietary habits to adapt to the special plateau environment. Colonoscopy exams of more than 6,000 native Tibetans showed that they have much lower rates of intestinal polyps, adenoma, colon cancer, and other intestinal diseases, compared to people who live at lower altitudes, especially in the coastal areas of China or in western countries [14]. Therefore, a better understanding of the gut microbiota of native Tibetans may help us better understand the adaption of Tibetans to the high altitude and their overall health.

Metagenomic analysis, including 16S rRNA sequence analysis, of human gut microbiota has revealed many functional roles of the gut microbiota to human hosts [15, 16]. Using the 16S rRNA sequencing technique, we previously showed that native Tibetans have an enriched gut microbiota, including *Enterococcaceae*, *Prevotellaceae*, *Prevotella*, and *Enterococcus*, in comparison to other populations [17]. We also showed the effects of the hypoxic environment on the gut microbial composition in Hans and Tibetans and compared the difference of gut microbial components between low altitude Hans and high altitude Hans in our previous study [17]. In this study, we compared gut microbiota between healthy native Tibetans and Hans living at the same altitude by high-throughput sequencing of total genomic DNA from fecal samples. The goal of our study was to better understand the role of different genetic factors (race) and environmental factors (diets and consumption of antibiotics) in shaping the composition and function of gut microbiota in populations living at high altitudes.

## 2. Methods

### 2.1. Participant Information

The participants in this study included 15 native Tibetans and 12 Hans. The Han population migrated to and has lived in Lhasa for over 10 years. The Tibetans included 8 men and 7 women between 32 and 44 years old. The Hans included 6 men and 6 women between 33 and 43 years old. Physical examination and medical records in the People's Hospital of Tibet Autonomous Region showed that the participants had no bowel diseases or metabolic diseases and had not taken any antibiotics within 2 months prior to sample collection. The native Tibetans followed the traditional lifestyle and dietary habits of their ethnic group. Meat, Zanba (a roasted barley flour), and dairy products formed their major diet, while few vegetables and fruits were consumed. The Hans followed their traditional dietary habits, which included wheat, rice, and more vegetables/fruits. The study was approved by the Ethics Committee of Guangzhou First People's Hospital of Guangzhou Medical University. Written informed consent was obtained from all individual participants prior to the study.

### 2.2. Sample Collection and DNA Isolation

Approximately 20 g fresh fecal samples were collected in sterilized 10 mL centrifuge tubes. All samples were collected by trained doctors from the First People's Hospital of Guangzhou and the People's Hospital of Tibet Autonomous Region. DNA in fecal samples was extracted using the E.Z.N.A. Stool DNA Kit (Omega, USA) according to the manufacturer's instructions.

### 2.3. DNA Library Construction, DNA Sequencing, and Quality Control

DNA libraries were constructed with a mean insert size of 300 bases using the TruSeq™ DNA Sample Prep Kit according to the manufacturer's instruction (Illumina, USA). The libraries were amplified by polymerase chain reaction (PCR) and sequenced using 100 base paired-end reads on the Illumina HiSeq System (Illumina); the reads were deposited into the NCBI short read archive database (SRP199009). The high quality reads after quality control were used for further analysis, including all abundance analysis.

### 2.4. Data Analysis

Basic Local Alignment Search Tool (BLAST) analysis was conducted using BLAST Version 2.2.28+ (http://blast.ncbi.nlm.nih.gov/Blast.cgi). Microbial species were determined by BLAST analysis of the clean reads against the small subunit rRNA (SSU rRNA) database (https://www.arb-silva.de) [18, 19].

Principal component analysis (PCA) was performed based on the composition of the bacterial communities as described previously [20]. Principal coordinate analysis (PCoA) was performed to further compare the abundance of microbiota between the two populations as described previously [21].

Contigs were constructed using SOAPdenovo software (version 1.3) [22] (http://soap.genomics.org.cn). Contigs longer than 100 bp were then used to predict open reading frames (ORFs) using MetaGeneAnnotator [23] (http://metagene.cb.k.u-tokyo.ac.jp). The nonredundant ORFs, as well as the clusters of nonredundant ORFs, were predicted using CD-HIT software [24] (http://www.bioinformatics.org/cd-hit). The abundance of nonredundant ORFs was calculated using SOAPaligner software (http://soap.genomics.org.cn/).

Clusters of orthologous groups (COGs) were predicted based on ORFs using BLAST analysis against the eggNOG database (evolutionary genealogy of genes: Non-supervised Orthologous Groups, http://eggnog.embl.de).

Kyoto Encyclopedia of Genes and Genomes (KEGG) pathways and modules were predicted by BLAST analysis of the high quality reads against the KEGG database.

The abundance of carbohydrate-active enzymes was analyzed by comparing the clean reads to the carbohydrate-active enzyme database (http://www.cazy.org) using hmmscan software (http://hmmer.janelia.org/search/hmmscan). The antibiotic resistance genes were predicted using BLAST analysis against the antibiotic resistance gene database [25] (http://ardb.cbcb.umd.edu).

### 2.5. Statistical Analysis

Wilcoxon rank-sum test was used to compare the statistical differences between the microbiota of the two populations. Both a *P* value < 0.05 and a *q* value (false discovery rate, FDR) < 0.01 were considered statistically significant.

## 3. Results

### 3.1. Baseline Characteristics of Participants

The baseline and physical characteristics of the study population are listed in Table 1. There were significant differences for the dietary habits between native Tibetans and Hans (*P* < 0.01). In contrast, no significant difference was found for other parameters including age, gender, and BMI (*P* > 0.05).

### 3.2. The Microbiota Abundance Was Higher in Native Tibetans than in Hans

After removing low quality reads and host sequences, averages of 22,407,937 and 30,918,028 high quality reads were obtained from the fecal DNA samples of native Tibetans and Hans, respectively ([Supplementary-material supplementary-material-1]). BLAST analysis showed that 99.17% of the sequences in Hans and 99.83% in Tibetans were bacteria. Due to the extreme small percentages of the nonbacterial species, only bacteria were further analyzed.

**Figure 1:**
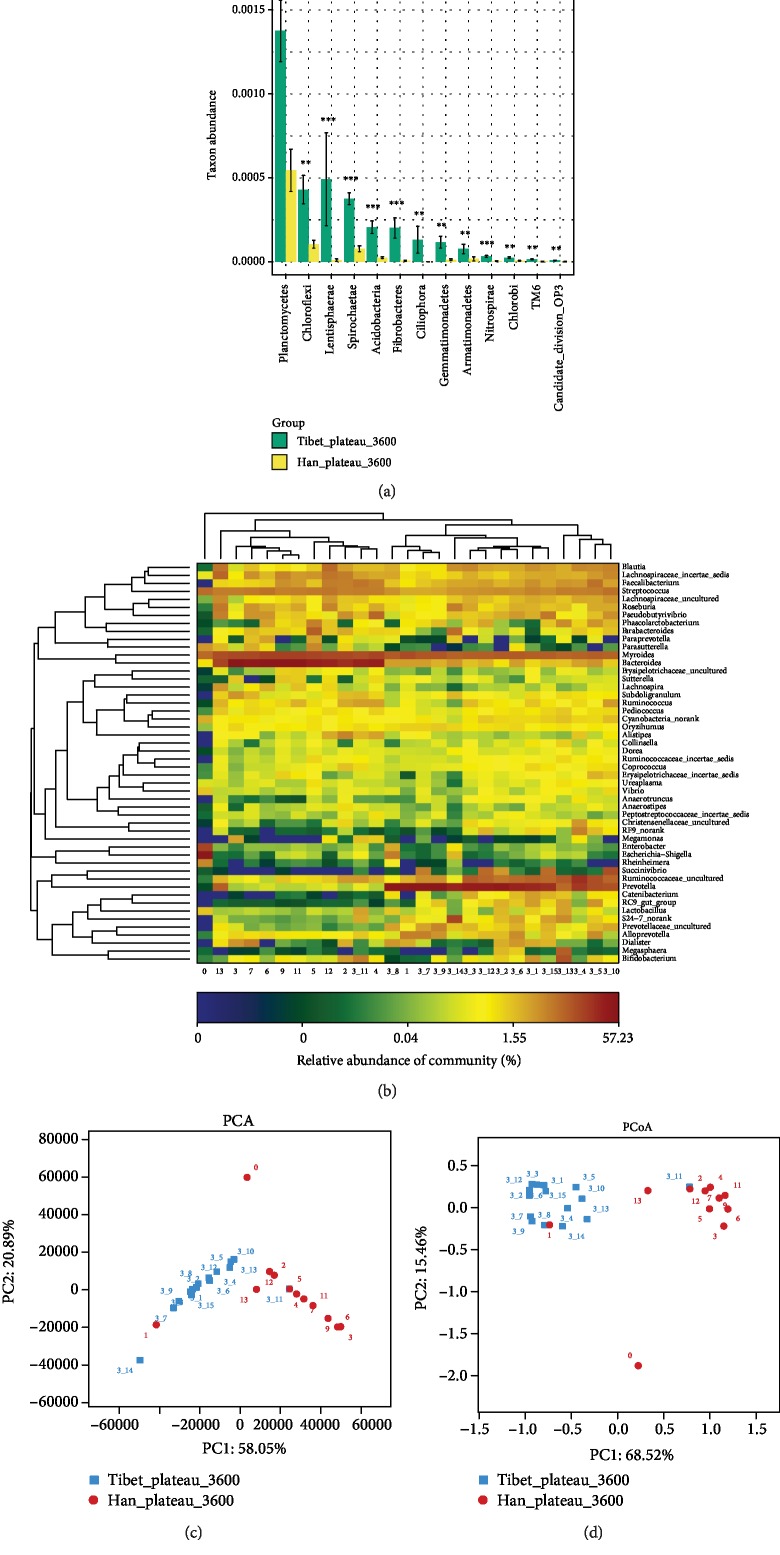
Comparison of the gut microbiota between the native Tibetans and the Chinese Hans at the phylum and genus levels. (a) Wilcoxon rank-sum analysis showing the significantly different gut microbiota phyla between the native Tibetans and the Hans. ^∗∗∗^*P* < 0.001, ^∗∗^*P* < 0.01, and ^∗^*P* < 0.05. (b) Heat map showing the most relatively abundant 50 genera of the gut microbiota of both the native Tibetans and the Hans. Sample ID 0-13: samples of Hans; sample ID 3_1-3_15: samples of native Tibetans. (c) Principal component analysis (PCA) showing differences in the gut microbiota between the native Tibetans and the Hans based on the composition of the bacterial communities. (d) Principal coordinate analysis (PCoA) of the differences in the gut microbiota between the native Tibetans and the Hans based on the distance matrix of Bray-Curtis dissimilarity of the microbial community.

Wilcoxon rank-sum test showed that many of the microbes from the two populations differed significantly at both phylum (Figure 1(a)) and genus levels ([Supplementary-material supplementary-material-1]). The *Planctomycetes* was the most abundant phyla both in native Tibetans and in Hans. Furthermore, the most relatively abundant phyla for native Tibetans were *Bacteroidetes* (15.66%), *Firmicutes* (11.10%), *Proteobacteria* (1.32%), *Actinobacteria* (1.10%), and *Tenericutes* (0.35%), while the most relatively abundant phyla for Hans were *Bacteroidetes* (16.28%), *Firmicutes* (8.41%), *Proteobacteria* (2.93%), *Actinobacteria* (0.49%), and *Cyanobacteria* (0.21%). A total of 1,625 genera were identified, among which 1002 genera were shared between Tibetans and Hans, but 217 genera were in Tibetans only and 406 genera were in Hans only ([Supplementary-material supplementary-material-1]). The detailed genus distribution is listed in [Supplementary-material supplementary-material-1].

The microbiota abundance of more than 130 genera was found to differ significantly between the Tibetans and Hans (*P* < 0.01) ([Supplementary-material supplementary-material-1]). The abundance of the majority of genera was significantly higher in Tibetans than in Hans (*P* < 0.01). Only three genera, including *Myroides*, *Nitrosomonadaceae_uncultured*, and *SC-I-84_norank*, were more abundant in Hans than in Tibetans (*P* < 0.01) ([Supplementary-material supplementary-material-1]). Interestingly, *Myroides* was found at high levels in both Hans and Tibetans.

We compared the relative distribution of the top 50 prevalent genera. As shown in Figure 1(b), a clear distinction was observed between the two populations. The relative abundance in native Tibetans was higher in general, except for a few genera, such as *Bacteroides*, *Myroides*, and *Alloprevotella*. PCA also showed that the microbiota of the two populations clearly differed (Figure 1(c)). PCoA showed a very similar pattern to PCA (Figure 1(d)). Interestingly, in both PCA and PCoA analyses, sample 1 of Hans was more closely aligned with the native Tibetans. And we also found sample 0 of Hans was an obvious outlier by both analyses (Figures 1(c) and 1(d)).

### 3.3. The Number of Microbial Genes Was Higher in the Native Tibetans than in the Hans

The number of microbial genes was 4.9 times higher in Tibetans than in Hans. Based on the high quality reads ([Supplementary-material supplementary-material-1]), the numbers of contigs and ORFs were calculated (Table 2). The average numbers of contigs and ORFs were all higher in the native Tibetans than in the Hans. However, the average lengths of ORFs in the native Tibetans were much shorter than those in the Hans (Table 2 and [Supplementary-material supplementary-material-1]).

**Figure 2:**
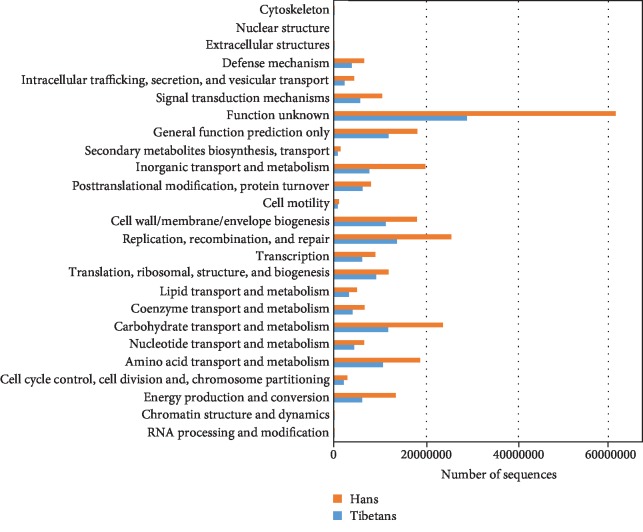
Comparison of the relative abundance (%) of the clusters of orthologous groups (COGs) in the gut microbiota between the native Tibetans and the Hans.

The identified genes were further analyzed according to the COGs, which are classified into different functional categories. As shown in Figure 2, the relative abundance of COGs was much higher in the gut microbiota of the Hans than in the native Tibetans (*P* = 0.002). The top 10 most abundant COGs in each ethnic group are listed in Table 3 (Hans) and Table 4 (Tibetans).

### 3.4. The Abundance of Metabolic Pathways and Modules Differed Significantly between the Native Tibetans and the Hans

**Figure 3:**
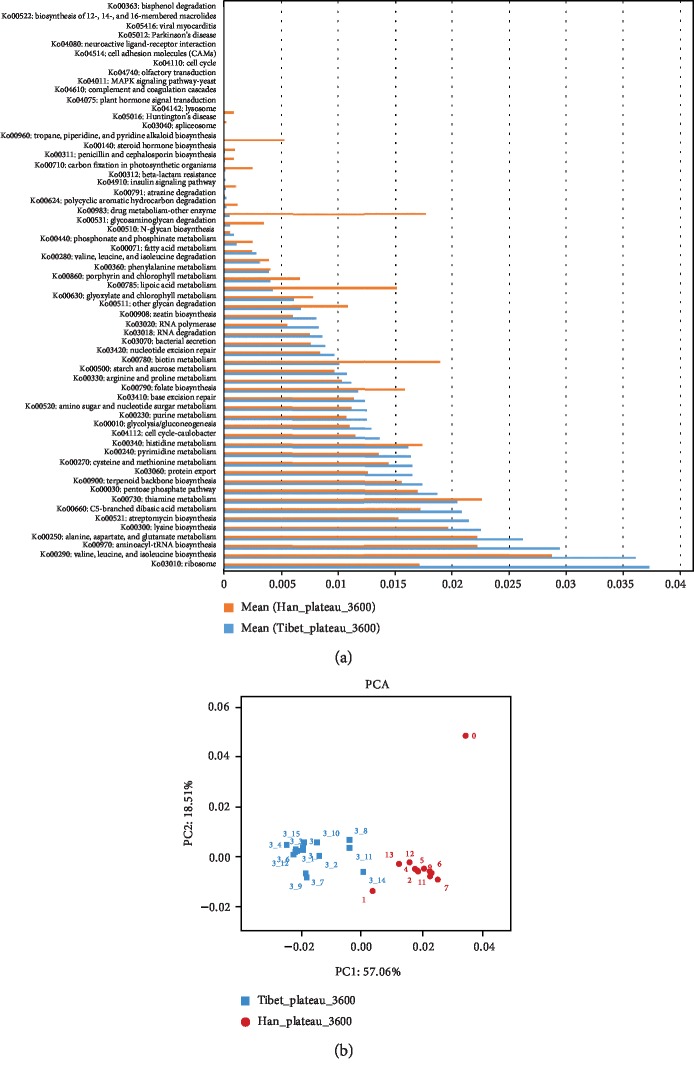
Comparison of the abundances of KEGG pathways and modules in the gut microbiota between the native Tibetans and the Hans. (a) Significantly different KEGG pathways between the two populations (*P* < 0.05). (b) PCA analysis of the abundance of KEGG pathways between the native Tibetans and the Hans.

The abundance of 61 metabolic pathways was identified to be significantly different between the native Tibetans and Hans (*P* < 0.05, Figure 3(a)). The most abundant metabolic pathway in native Tibetans was ribosome (KO03010). The most abundant metabolic pathway in the Hans was the valine, leucine, and isoleucine biosynthesis (KO00290), which is involved in the biosynthesis and metabolism of these amino acids. Some of these most significantly different pathways were among the top 50 abundant metabolic pathways in native Tibetans ([Supplementary-material supplementary-material-1]a). PCA of the abundance of metabolic pathways further confirmed that the differences between the two populations were significant (PC1: 57.06% and PC2: 18.51%) (Figure 3(b)).

In addition, Wilcoxon rank-sum test showed that 12 modules were significantly different between the two populations (*P* < 0.05). All modules, except module M00178 (bacteria ribosome that is involved in RNA/protein processing), were more abundant in the Han population than in the native Tibetans (*P* < 0.05) ([Supplementary-material supplementary-material-1]).

### 3.5. The Abundance of Carbohydrate-Active Enzyme Modules Was Higher in the Hans than in the Native Tibetans

**Figure 4:**
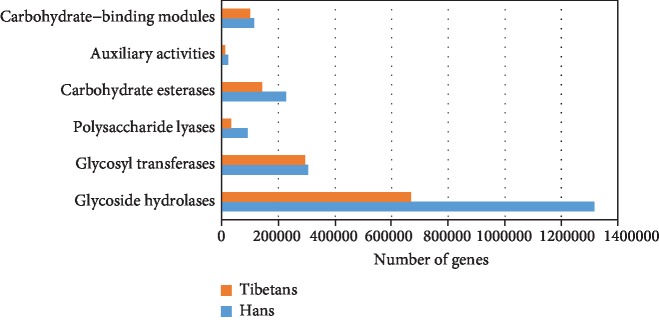
Comparison of the abundance of carbohydrate-active enzyme modules between the native Tibetans and the Hans.

To compare the differences of carbohydrate-active enzymes between the native Tibetans and Hans, the identified genes were compared against the database of carbohydrate-active enzymes [26] (http://www.cazy.org), and the relative abundance of the carbohydrate-active enzyme module was calculated. The abundance of carbohydrate-active enzyme modules is significantly higher in the Hans than in the native Tibetans (*P* < 0.05). And the most abundant enzyme module for both the native Tibetans and the Hans was glycoside hydrolases, followed by glycosyltransferases (Figure 4).

### 3.6. Antibiotic Resistance Gene Abundance Was Higher in the Hans than in the Native Tibetans

**Figure 5:**
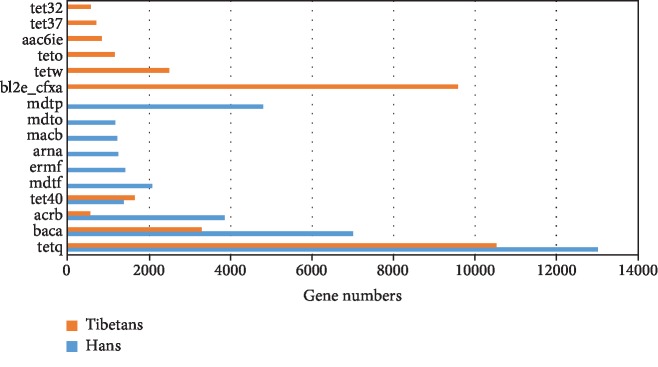
Comparison of the top 10 most abundant antibiotic resistance gene types present in the gut microbiota of the native Tibetans and Hans.

Using the Antibiotic Resistance Genes Database (ARDB, http://ardb.cbcb.umd.edu), we analyzed the relative abundances of antibiotic resistance genes in the gut microbiota of the native Tibetans and Hans. Among the 373 types of antibiotic resistance genes available in the database, we identified 56 types in the Han samples ([Supplementary-material supplementary-material-1]) and 50 in the native Tibetan samples ([Supplementary-material supplementary-material-1]). In addition, the average abundance of the antibiotic resistance genes was significantly higher in the Hans (910) than in the native Tibetans (676) (*P* < 0.05).  Figure 5 shows the top 10 most abundant antibiotic resistance gene types in both the Hans and the native Tibetans. Four of 10 resistance gene types were shared between the two populations and the other 6 differed. The 4 common antibiotic resistance gene types included tetq (a ribosomal protection protein that protects the ribosome from the translation inhibition of tetracycline), baca (an undecaprenyl pyrophosphate phosphatase that is resistant to bacitracin), acrb (a multidrug resistance efflux pump), and tet40 (a tetracycline efflux pump). Three of the four gene types (tetq, baca, and acrb) were found to be present at significantly higher levels in Hans than in native Tibetans (*P* < 0.05) (Figure 5). In addition, the 4 resistance gene types were found in all 12 Han samples and all 15 native Tibetan samples, suggesting that bacitracin and tetracycline are among the most commonly used antibiotics in these populations.

## 4. Discussion

In this study, the numbers of microbial species (at both phylum and genus levels) and numbers of genes were greater in Tibetans. The difference of average ORF length in the two groups may be the possible reason for this result. Additionally, the gene copy numbers in each phylum were higher in the Hans than in the native Tibetans. This also explains, in part, the higher number of high quality reads in the Han population. It is not clear why the gene length was shorter in the Tibetan samples. It is possible that DNAs from different samples have different sensitivities to PCR amplification and/or the sequencing method [27]. We can also speculate that this is a result of different dietary habits between native Tibetan and Hans.

It has been reported that the *Bacteroides* species are dominant in people who consume more proteins and animal fats, whereas the *Prevotella* species, which are rich in genes for cellulose and xylan hydrolysis, are predominant in those who eat more carbohydrates and fibers [9]. Interestingly, we found that the genus *Prevotella* was most abundant in native Tibetans, while *Bacteroides* was the most abundant genus in the Hans. However, the native Tibetans normally consume more proteins (meat and dairy products), while the Hans consume more carbohydrates (wheat and rice) and vegetables. The abundance of *Bacteroides* and *Prevotella* in native Tibetans and Hans was apparently different from previous reports [9]. We speculate that high altitude, or even urbanization or industrialization, may contribute to this difference. For example, *Prevotella* was found to make up more than 50% of the gut bacteria in children from rural Africa but was absent in children living in more industrialized Europe [10]. A similar study showed that *Prevotella* was predominant in native Africans who lived in rural areas and *Bacteroides* was more abundant in urbanized African Americans [28]. It is proposed that people of the rural communities who maintain natural diets and consume less industrial-type food and fewer antimicrobial agents may possess healthier microbiota [29, 30]. The native Tibetans may provide another example of rural microbiota. Further study of the genes and gene functions in these two bacterial genera could help us better understand differences of microbiota and their physiological functions in human hosts.

In the present study, we also found that *Myroides* was the second most abundant genus in Hans and the third most abundant genus in native Tibetans. *Myroides* is not a common bacterial genus found in human gut microbiota but rather is commonly found in the environment [31]. Also, *Myroides* was reported to be associated with chronic obstructive pulmonary disease and pneumonia [32], which may explain why people who live in a dry environment at high altitude have a higher incidence of pulmonary diseases [15].

In our study, we found that many metabolic pathways differed greatly between the gut microbiota of the two populations. For example, the tropane, piperidine, and pyridine alkaloid biosynthesis pathway, which is required for metabolism and biosynthesis of other secondary metabolites, was significantly different. The abundance of this pathway was significantly higher in the Hans than in the native Tibetans, suggesting that microbes in Hans have a higher capacity for synthesizing secondary metabolites.

Studies have shown that antibiotic usage affects the assembly of the microbiota and is associated with increased risks of diseases [33, 34]. A recent study even showed that antibiotic use accelerates the development of type 1 diabetes due to perturbation of the gut microbiome [35]. The unnecessary use of antibiotics could also act as a selective pressure for bacteria to increase the frequency of antibiotic resistance genes [36]. In addition to natural selection, genetic materials can be transmitted through horizontal gene transfer (HGT), a process of transferring genetic information between two living organisms [37]. HGT allows new bacterial variants to arise without a mutation and is a major pathway for the spread of antibiotic resistance in bacteria. In the current study, we showed that both the number and the abundance of antibiotic resistance genes were significantly higher in the gut microbiota of the Hans than in the native Tibetans, indicating that the Hans use antibiotics more frequently. Importantly, we speculate that the heavy use of antibiotics may also contribute the lower numbers of microbial species and genes. In addition, we found that the abundance of the carbohydrate-active enzyme modules was higher in the Hans than in the native Tibetans. This reflects the differences in dietary habits between the two populations; the Hans consume more carbohydrates than the Tibetans. It may also be one of the causes of the differences in the microbiota.

## Figures and Tables

**Table 1:** The baseline characteristics of all participants.

Characteristics	Native Tibetans	Hans	*P* value
Subjects (*n*)	15	12	—
Gender (M/F) (*n* (%))	8/7 (53.3%)	6/6 (50.0%)	0.86
Mean age (range) (years)	37.7 ± 3.8 (32-44)	38.1 ± 3.2 (33-43)	0.38
BMI (range)	30.8 ± 2.1 (28-34)	31.8 ± 2.3 (29-36)	0.96
Dietary habits (Tibetans' traditional habit/Hans' traditional habit)	15/0	0/12	<0.01
Drinking (no/yes)	9/6	8/4	0.72
Smoking (no/yes)	8/7 (53.3%)	6/6 (50.0%)	0.86
Frequency of food (per day)	3 times	3 times	—
Sleep habits	Day	Day	—
Mental stress	Social	Social	—
Diet and sleep timings	Regular	Regular	—

BMI: body mass index; Tibetans' traditional habit: meat, Zanba (a roasted barley flour), and dairy products formed their major diet; Hans' traditional habit: wheat, rice, and more vegetables/fruits formed their major diet.

**Table 2:** Average numbers of contigs, ORFs, and nonredundant ORFs in the gut microbiota of the native Tibetans and the Hans.

	Hans		Tibetans	
	Average no.	Average length (bps)	Average no.	Average length (bps)
Contigs	278,303.3	2038.6	380,198.2	1744.5
ORFs (>100 bps)	99,587.3	775.0	217,017.1	383.2
Nonredundant ORFs	41,329.6	794.2	149,662.0	388.8

ORF: open reading frames.

**Table 3:** Top 10 abundant COGs of the gut microbiota in the Hans.

#COG	Sum (%)	Description
NOG00099	2,936,314 (19.64)	TonB-dependent receptor plug
COG3250	2,051,906 (13.72)	Hydrolase family 2
COG0841	1,867,100 (12.49)	Acriflavin resistance protein
COG0534	1,378,376 (9.22)	Mate efflux family protein
COG1472	1,338,522 (8.95)	Hydrolase family 3
COG0550	1,248,452 (8.35)	Releases the supercoiling and torsional tension of DNA, which is introduced during the DNA replication and transcription, by transiently cleaving and rejoining one strand of the DNA duplex. Introduces a single-strand break via transesterification at a target site in duplex DNA. The scissile phosphodiester is attacked by the catalytic tyrosine of the enzyme, resulting in the formation of a DNA-(5′-phosphotyrosyl)-enzyme intermediate and the expulsion of a 3′-OH DNA strand. The free DNA strand then undergoes passage around the unbroken strand, thus removing DNA supercoils. Finally, in the religation step, the DNA 3′-OH attacks the covalent intermediate to expel the active-site tyrosine and restore the DNA phosphodiester backbone (by similarity)
COG0642	1,113,768 (7.45)	Histidine kinase
NOG00011	1,074,890 (7.19)	Histidine kinase
COG0827	1,063,196 (7.11)	DNA methylase
COG4646	878,308 (5.87)	Helicase

COG: clusters of orthologous group.

**Table 4:** Top 10 abundant COGs of the gut microbiota in native Tibetans.

#COG	Sum (%)	Description
COG1373	1,412,968 (20.35)	ATPase (AAA+superfamily)
NOG00099	850,664 (12.25)	TonB-dependent receptor plug
NOG02827	780,632 (11.24)	ATPase (AAA)
COG0642	739,750 (10.65)	Histidine kinase
COG3250	654,486 (9.43)	Hydrolase family 2
COG0463	568,870 (8.19)	Glycosyltransferase, family 2
COG1472	533,440 (7.68)	Hydrolase family 3
COG0793	474,230 (6.83)	Protease
COG0534	473,946 (6.83)	Mate efflux family protein
COG0745	454,886 (6.55)	DNA-binding response regulator, OmpR family

COG: clusters of orthologous group.

## Data Availability

The data used to support the findings of this study are available from the corresponding author upon request.

## Supplementary Materials

Supplementary Materials Figure S1: number of total bacterial genera in native Tibetans and Han Chinese. Figure S2: distribution of length of genes of the gut microbiota. (a) Gene length distribution in the Hans. (b) Gene length distribution in the native Tibetans. Figure S3: (a) heat map of the top 50 most abundant KEGG pathways in both the native Tibetans and Han Chinese (sample IDs 3_1 to 3_15 indicate the 15 individual samples). (b) Difference in the abundance of the KEGG modules between the native Tibetans and the Hans. Table S1: quality control of the sequence reads. Table S2: genera of gut microbiota that are significantly different between the native Tibetans and the Chinese Hans. Table S3: bacterial genera distribution in native Tibetans and Han Chinese. Table S4A: antibiotic-resistant genes identified in gut microbiota from the Han samples. Table S4B: antibiotic-resistant genes identified in gut microbiota from the Tibetan samples.

## References

[1] Nicholson J. K., Holmes E., Kinross J. (2012). Host-gut microbiota metabolic interactions. *Science*.

[2] Ottman N., Smidt H., de Vos W. M., Belzer C. (2012). The function of our microbiota: who is out there and what do they do?. *Frontiers in Cellular and Infection Microbiology*.

[3] Gill S. R., Pop M., Deboy R. T. (2006). Metagenomic analysis of the human distal gut microbiome. *Science*.

[4] Belkaid Y., Hand T. W. (2014). Role of the microbiota in immunity and inflammation. *Cell*.

[5] Nagpal R., Yadav H., Marotta F. (2014). Gut microbiota: the next-gen frontier in preventive and therapeutic medicine?. *Frontiers in Medicine*.

[6] Lucchese M., Scopinaro N. (2015). *Minimally Invasive Bariatric and Metabolic Surgery*.

[7] Claesson M. J., Cusack S., O'Sullivan O. (2011). Composition, variability, and temporal stability of the intestinal microbiota of the elderly. *Proceedings of the National Academy of Sciences of the United States of America*.

[8] Yatsunenko T., Rey F. E., Manary M. J. (2012). Human gut microbiome viewed across age and geography. *Nature*.

[9] David L. A., Maurice C. F., Carmody R. N. (2014). Diet rapidly and reproducibly alters the human gut microbiome. *Nature*.

[10] De Filippo C., Cavalieri D., Di Paola M. (2010). Impact of diet in shaping gut microbiota revealed by a comparative study in children from Europe and rural Africa. *Proceedings of the National Academy of Sciences of the United States of America*.

[11] Schnorr S. L., Candela M., Rampelli S. (2014). Gut microbiome of the Hadza hunter-gatherers. *Nature Communications*.

[12] Zhang J., Guo Z., Xue Z. (2015). A phylo-functional core of gut microbiota in healthy young Chinese cohorts across lifestyles, geography and ethnicities. *The ISME Journal*.

[13] Li L., Zhao X. (2015). Comparative analyses of fecal microbiota in Tibetan and Chinese Han living at low or high altitude by barcoded 454 pyrosequencing. *Scientific Reports*.

[14] Ciren Y., Deji D., Li K. (2016). Colonoscopy analysis of 6324 Tibetans patients. *Chinese Journal of Digestive Endoscopy*.

[15] Wang M., AhrnÃ© S., Jeppsson B., Molin G Ã.¶r. (2005). Comparison of bacterial diversity along the human intestinal tract by direct cloning and sequencing of 16S rRNA genes. *FEMS Microbiology Ecology*.

[16] Gosalbes M. J., Durbán A., Pignatelli M. (2011). Metatranscriptomic approach to analyze the functional human gut microbiota. *PLoS One*.

[17] Li K., Dan Z., Gesang L. (2016). Comparative analysis of gut microbiota of native Tibetan and Han populations living at different altitudes. *PLoS One*.

[18] Cantarel B. L., Coutinho P. M., Rancurel C., Bernard T., Lombard V., Henrissat B. (2009). The Carbohydrate-Active EnZymes database (CAZy): an expert resource for Glycogenomics. *Nucleic Acids Research*.

[19] Turnbaugh P. J., Hamady M., Yatsunenko T. (2009). A core gut microbiome in obese and lean twins. *Nature*.

[20] Ou J., Carbonero F., Zoetendal E. G. (2013). Diet, microbiota, and microbial metabolites in colon cancer risk in rural Africans and African Americans. *The American Journal of Clinical Nutrition*.

[21] Tyakht A. V., Kostryukova E. S., Popenko A. S. (2013). Human gut microbiota community structures in urban and rural populations in Russia. *Nature Communications*.

[22] Tyakht A. V., Alexeev D. G., Popenko A. S., Kostryukova E. S., Govorun V. M. (2014). Rural and urban microbiota: to be or not to be?. *Gut Microbes*.

[23] Endicott-Yazdani T. R., Dhiman N., Benavides R., Spak C. W. (2015). Myroides odoratimimus bacteremia in a diabetic patient. *Baylor University Medical Center Proceedings*.

[24] Benedetti P., Rassu M., Pavan G., Sefton A., Pellizzer G. (2011). Septic shock, pneumonia, and soft tissue infection due to Myroides odoratimimus: report of a case and review of Myroides infections. *Infection*.

[25] Quast C., Pruesse E., Yilmaz P. (2013). The SILVA ribosomal RNA gene database project: improved data processing and web-based tools. *Nucleic Acids Research*.

[26] Yilmaz P., Parfrey L. W., Yarza P. (2014). The SILVA and "All-species Living Tree Project (LTP)" taxonomic frameworks. *Nucleic Acids Research*.

[27] Schloss P. D., Westcott S. L., Ryabin T. (2009). Introducing mothur: open-source, platform-independent, community-supported software for describing and comparing microbial communities. *Applied and Environmental Microbiology*.

[28] Bronstein A. M., Bronstein M. M., Kimmel R. (2006). Generalized multidimensional scaling: a framework for isometry-invariant partial surface matching. *Proceedings of the National Academy of Sciences of the United States of America*.

[29] Luo R., Liu B., Xie Y. (2012). SOAPdenovo2: an empirically improved memory-efficient short-read de novo assembler. *Gigascience*.

[30] Noguchi H., Taniguchi T., Itoh T. (2008). MetaGeneAnnotator: detecting species-specific patterns of ribosomal binding site for precise gene prediction in anonymous prokaryotic and phage genomes. *DNA Research*.

[31] Li W., Godzik A. (2006). Cd-hit: a fast program for clustering and comparing large sets of protein or nucleotide sequences. *Bioinformatics*.

[32] Liu B., Pop M. (2009). ARDB--Antibiotic Resistance Genes Database. *Nucleic Acids Research*.

[33] Marra F., Marra C. A., Richardson K. (2009). Antibiotic use in children is associated with increased risk of asthma. *Pediatrics*.

[34] Modi S. R., Collins J. J., Relman D. A. (2014). Antibiotics and the gut microbiota. *The Journal of Clinical Investigation*.

[35] Livanos A. E., Greiner T. U., Vangay P. (2016). Antibiotic-mediated gut microbiome perturbation accelerates development of type 1 diabetes in mice. *Nature Microbiology*.

[36] Kolar M., Urbanek K., Latal T. (2001). Antibiotic selective pressure and development of bacterial resistance. *International Journal of Antimicrobial Agents*.

[37] de la Cruz F., Davies J. (2000). Horizontal gene transfer and the origin of species: lessons from bacteria. *Trends in Microbiology*.

