# Supplementary material for: Host Genetic and Environmental Factors Shape the Composition and Function of Gut Microbiota in Populations Living at High Altitude
Source: Biomed Res Int. 2020 Feb 28;2020:1482109. doi: 10.1155/2020/1482109 (PMC7071804; doi:10.1155/2020/1482109)
Supplement: Supplementary Materials — Figure S1: number of total bacterial genera in native Tibetans and Han Chinese. Figure S2: distribution of length of genes of the gut microbiota. (a) Gene length distribution in the Hans. (b) Gene length distribution in the native Tibetans. Figure S3: (a) heat map of the top 50 most abundant KEGG pathways in both the native Tibetans and Han Chinese (sample IDs 3_1 to 3_15 indicate the 15 individual samples). (b) Difference in the abundance of the KEGG modules between the native Tibetans and the Hans. Table S1: quality control of the sequence reads. Table S2: genera of gut microbiota that are significantly different between the native Tibetans and the Chinese Hans. Table S3: bacterial genera distribution in native Tibetans and Han Chinese. Table S4A: antibiotic-resistant genes identified in gut microbiota from the Han samples. Table S4B: antibiotic-resistant genes identified in gut microbiota from the Tibetan samples. [file 1482109.f1.pdf]

Table S1. Quality control of the sequence reads

Sample Id	Raw Reads	Low Quality Reads%	Host Reads%	High quality
**3_1**	55,858,356	70.86%	0.33%	16,223,028
**3_2**	51,757,504	30.48%	0.19%	35,912,596
**3_3**	53,134,418	66.01%	9.41%	16,358,646
**3_4**	64,619,834	69.33%	0.24%	18,605,339
**3_5**	61,227,976	68.59%	0.01%	19,228,180
**3_6**	54,811,970	66.56%	0.29%	18,276,164
**3_7**	65,786,596	66.76%	0.26%	21,807,526
**3_8**	49,714,782	27.90%	0.33%	35,725,452
**3_9**	54,485,568	64.84%	0.02%	19,150,586
**3_10**	45,724,010	26.68%	0.02%	33,519,184
**3_11**	62,900,014	60.78%	23.80%	18,799,058
**3_12**	58,154,640	57.17%	0.04%	24,900,122
**3_13**	64,618,362	71.11%	0.03%	18,664,044
**3_14**	54,091,108	83.04%	18.64%	7,462,566
**3_15**	72,294,564	56.42%	0.07%	31,486,562
**S0**	46,189,622	13.38%	3.03%	38,794,848
**S1**	36,638,842	8.39%	0.01%	33,561,978
**S2**	36,263,776	13.22%	0.03%	31,460,040
**S3**	38,792,676	10.45%	0.07%	34,715,240
**S4**	38,125,376	11.64%	0.01%	33,682,404
**S5**	36,806,512	7.57%	0.04%	34,007,586
**S6**	36,862,596	10.25%	0.10%	33,052,238
**S7**	34,003,480	9.94%	0.02%	30,615,974
**S9**	33,642,370	13.71%	0.15%	28,985,416
**S11**	28,328,570	23.36%	0.05%	21,699,634
**S12**	26,114,462	21.55%	0.01%	20,483,936
**S13**	36,423,894	17.74%	0.01%	29,957,042

Note: Sample IDs 3_1 to 3_15 indicate the sample numbers of native Tibetans. S0 to S13 indicate the sample numbers of Chinese Hans.

Table S2. Genera of gut microbiota that are significantly different between the native Tibetans and Chinese Hans

Genus	Relative abundance (%) (Tibet)	sd (Tibet)	Relative abundance (%) (Han)	sd (Han)	p-value	q-value
Filifactor	2.39E-05	2.01E-05	1.35E-06	4.69E-06	0.000104	0.000959
Parvimonas	0.000111	0.000185	7.31E-06	8.50E-06	0.000118	0.001033
Arcicella	2.34E-05	2.28E-05	0	0	0.000127	0.001033
Clostridium_sensu_stricto_7	2.21E-05	2.91E-05	0	0	0.000127	0.001033
Dasania	1.40E-05	1.17E-05	0	0	0.000127	0.001033
Ureibacillus	0.000201	0.000269	0	0	0.000127	0.001033
Butyrivibrio	0.00224	0.002412	0.00015	0.000324	0.000128	0.001033
Marinilabiaceae_uncultured	0.000567	0.000292	0.000177	0.000122	0.000128	0.001033
Algoriphagus	6.39E-05	6.47E-05	1.53E-06	3.59E-06	0.000142	0.001113
Gelria	5.86E-05	2.87E-05	8.81E-06	2.59E-05	0.000143	0.001113
Anaerosporobacter	0.00025	0.000118	5.90E-05	6.31E-05	0.000156	0.001173
Tannerella	0.001481	0.000913	0.000422	0.000501	0.000156	0.001173
vadinBB60_norank	0.001581	0.001604	8.69E-05	0.000165	0.000181	0.001334
Candidatus_Arthromitus	0.000148	6.24E-05	3.37E-05	5.26E-05	0.000185	0.001334
Limnobacter	0.000131	7.37E-05	3.17E-05	2.99E-05	0.000189	0.001334
Syntrophococcus	0.00084	0.000465	0.000207	0.000196	0.000189	0.001334
Nitrospira	3.23E-05	2.19E-05	3.79E-06	6.05E-06	0.000212	0.001474
Atopostipes	4.34E-05	2.40E-05	3.64E-06	5.70E-06	0.000233	0.001591
Arthrobacter	6.20E-05	5.14E-05	1.38E-05	1.33E-05	0.000275	0.001841
Myroides	0.095802	0.011138	0.148277	0.027166	0.000278	0.001841
Desulfovibrionaceae_uncultured	5.11E-05	4.30E-05	6.03E-07	2.09E-06	0.000285	0.001863
Maritimimonas	1.52E-05	1.61E-05	0	0	0.00033	0.002125
Kurthia	3.53E-05	2.86E-05	4.10E-06	6.68E-06	0.000343	0.002178

Sporomusa	0.000108	9.95E-05	1.66E-05	2.13E-05	0.000387	0.00239
Fusibacter	3.92E-05	4.54E-05	1.48E-06	3.47E-06	0.000392	0.00239
Anaerolineaceae_uncultured	0.000209	0.000198	2.37E-05	2.51E-05	0.000404	0.00239
Prevotella	0.273326	0.155876	0.056298	0.161699	0.000404	0.00239
S24-7_norank	0.022241	0.039546	0.001709	0.004081	0.000404	0.00239
Tetrasphaera	0.000314	0.000376	6.59E-06	1.33E-05	0.000415	0.002427
BS11_gut_group_norank	0.000223	0.000267	1.55E-05	3.54E-05	0.000449	0.002586
JTB255_marine_benthic_group_norank	1.92E-05	1.60E-05	3.55E-06	6.06E-06	0.000457	0.002601
RB41_norank	5.56E-05	7.07E-05	1.42E-06	3.37E-06	0.000477	0.002659
Coriobacteriaceae_uncultured	0.000884	0.000732	0.000123	0.000123	0.000485	0.002659
Ruminococcaceae_uncultured	0.072807	0.052484	0.01226	0.009432	0.000485	0.002659
Nocardioides	0.000117	0.000146	5.49E-06	9.35E-06	0.000501	0.002715
Flammeovirgaceae_uncultured	1.41E-05	1.43E-05	8.08E-07	2.80E-06	0.00052	0.002779
Reichenbachiella	0.000148	0.000212	2.03E-06	5.05E-06	0.000578	0.003055
Aeriscardovia	0.000584	0.000985	7.50E-06	1.41E-05	0.000604	0.003153
Alpinimonas	0.000225	0.000143	3.43E-05	3.16E-05	0.000624	0.003189
Erysipelothrix	8.66E-05	6.24E-05	1.06E-05	1.48E-05	0.000625	0.003189
WCHB1-69_norank	2.60E-05	1.79E-05	6.13E-06	7.15E-06	0.000709	0.003576
Coxiella	1.19E-05	1.29E-05	0	0	0.000807	0.003786
Ekhidna	4.41E-05	5.18E-05	0	0	0.000807	0.003786
Marinicella	1.14E-05	1.03E-05	0	0	0.000807	0.003786
Sandaracinaceae_norank	2.23E-05	2.60E-05	0	0	0.000807	0.003786
Sporosarcina	8.10E-06	6.67E-06	0	0	0.000807	0.003786
Tissierella	1.45E-05	1.58E-05	0	0	0.000807	0.003786
Asteroleplasma	0.000104	0.000154	1.42E-06	4.92E-06	0.00081	0.003786

Bacteroides	0.039279	0.071934	0.307337	0.174107	0.00083	0.003841
SM2D12_norank	1.77E-05	1.93E-05	1.55E-06	3.71E-06	0.000843	0.00386
Mogibacterium	0.000317	0.000347	1.65E-05	1.52E-05	0.00089	0.004033
Comamonas	5.82E-05	4.73E-05	9.96E-06	1.79E-05	0.000946	0.004222
Selenomonas	0.000112	0.000174	1.60E-05	1.96E-05	0.000971	0.004222
Gastranaerophilales_norank	0.002395	0.00412	1.88E-06	6.53E-06	0.000982	0.004222
Parasutterella	0.000652	0.001997	0.011904	0.01487	0.000982	0.004222
Hydrogenoanaerobacterium	0.000152	8.99E-05	5.92E-05	4.64E-05	0.000987	0.004222
Family_XIII_uncultured	0.001578	0.000848	0.000467	0.00035	0.000989	0.004222
Subgroup_6_norank	8.50E-05	8.20E-05	8.13E-06	1.18E-05	0.001012	0.004278
TM6_norank	1.43E-05	9.34E-06	2.30E-06	5.47E-06	0.001218	0.005068
Saprospiraceae_uncultured	1.60E-05	1.34E-05	1.44E-06	3.39E-06	0.001221	0.005068
Mycoplasma	0.001085	0.000602	0.000416	0.000314	0.001393	0.005727
S0134_terrestrial_group_norank	8.04E-05	9.24E-05	8.36E-06	1.30E-05	0.001475	0.006008
Anaerotruncus	0.003244	0.002408	0.00073	0.001179	0.001648	0.006652
NB1-n_norank	0.000439	0.000875	6.72E-07	2.33E-06	0.001782	0.006903
Acetitomaculum	4.57E-05	3.20E-05	1.02E-05	1.61E-05	0.001844	0.006903
ABS-19_norank	5.25E-06	4.85E-06	0	0	0.001866	0.006903
Bryobacter	8.15E-06	9.50E-06	0	0	0.001866	0.006903
Cryomorpha	8.18E-06	8.82E-06	0	0	0.001866	0.006903
Desulfobulbus	9.12E-06	9.95E-06	0	0	0.001866	0.006903
Desulfomicrobium	1.92E-05	2.11E-05	0	0	0.001866	0.006903
Desulfurellaceae_uncultured	1.23E-05	1.32E-05	0	0	0.001866	0.006903
Vallitalea	9.37E-06	9.30E-06	0	0	0.001866	0.006903
possible_genus_04	7.62E-06	7.33E-06	0	0	0.001866	0.006903
Leifsonia	0.000468	0.000216	0.000205	0.000135	0.001945	0.007138
NS11-12_marine_group_norank	1.43E-05	1.23E-05	2.12E-06	5.13E-06	0.002074	0.007548

E01-9C-26_marine_group_norank	1.35E-05	1.22E-05	6.03E-07	2.09E-06	0.002143	0.007737
Epulopiscium	3.58E-05	3.66E-05	8.56E-06	1.27E-05	0.002178	0.007798
Flectobacillus	7.33E-05	5.64E-05	2.41E-05	1.88E-05	0.002287	0.00806
Paludibacter	0.000309	0.000604	5.60E-05	0.000123	0.002287	0.00806
Helcococcus	2.52E-05	3.46E-05	9.42E-07	3.26E-06	0.002572	0.00896
Ardenticatena	2.44E-05	2.31E-05	2.83E-06	5.45E-06	0.002583	0.00896
RF9_norank	0.007521	0.012161	0.000317	0.000686	0.002688	0.009251
Propionicicella	4.14E-07	1.60E-06	7.11E-06	7.13E-06	0.002778	0.009489
Nitrosomonadaceae_uncultured	4.17E-05	4.63E-05	0.000343	0.000398	0.002873	0.009736
Prochlorococcus	6.17E-05	4.08E-05	2.03E-06	4.98E-06	1.18E-05	0.000349
Tepidimicrobium	0.000124	7.74E-05	3.07E-06	4.60E-06	1.22E-05	0.000349
Caldicoprobacter	0.00077	0.000382	1.14E-05	1.04E-05	1.22E-05	0.000349
Marinilabiaceae_norank	0.00028	0.000196	1.11E-05	7.89E-06	1.24E-05	0.000349
Bacillus	0.000445	0.000179	8.69E-05	4.41E-05	1.26E-05	0.000349
Bacteroidales_uncultured	0.000975	0.000665	5.77E-05	3.06E-05	1.26E-05	0.000349
Cytophagaceae_uncultured	0.00088	0.00042	0.00025	9.83E-05	1.26E-05	0.000349
OPB54_norank	0.000524	0.00022	3.79E-05	2.95E-05	1.26E-05	0.000349
Porphyromonas	0.002601	0.001442	8.95E-05	4.80E-05	1.26E-05	0.000349
RC9_gut_group	0.011154	0.010858	2.97E-05	1.58E-05	1.26E-05	0.000349
vadinBC27_wastewater-sludge_group	0.001884	0.000857	7.23E-05	4.08E-05	1.26E-05	0.000349
Succinivibrio	0.023328	0.038285	3.74E-06	8.51E-06	1.36E-05	0.000356
Marinifilum	0.000384	0.000194	3.67E-05	2.98E-05	1.57E-05	0.000361
Catenibacterium	0.009005	0.008361	1.88E-05	2.99E-05	1.77E-05	0.000361
Paucimonas	5.11E-05	2.08E-05	6.03E-06	7.51E-06	1.77E-05	0.000361
Olivibacter	0.000143	8.51E-05	9.46E-06	1.05E-05	1.85E-05	0.000361
VC2.1_Bac22_norank	0.000113	5.83E-05	1.06E-05	1.33E-05	1.85E-05	0.000361

0319-6G20_norank	7.63E-05	4.44E-05	5.53E-06	1.00E-05	1.90E-05	0.000361
Oribacterium	0.001196	0.00098	5.74E-05	6.74E-05	1.95E-05	0.000361
Anaerococcus	9.92E-05	5.19E-05	7.34E-07	2.54E-06	2.24E-05	0.000383
Anaerovorax	4.73E-05	3.79E-05	7.34E-07	2.54E-06	2.24E-05	0.000383
SC-I-84_norank	0.000103	9.53E-05	0.000547	0.000366	3.02E-05	0.000487
NS9_marine_group_norank	7.60E-05	7.08E-05	1.41E-06	3.29E-06	3.07E-05	0.000487
Peptococcaceae_uncultured	0.000864	0.000621	5.43E-05	9.31E-05	3.70E-05	0.000552
Fastidiosipila	0.00017	0.000112	1.16E-05	1.00E-05	3.73E-05	0.000552
Ignatzschineria	7.42E-05	3.47E-05	7.99E-06	2.30E-05	4.49E-05	0.000583
AEGEAN-169_marine_group	4.53E-05	3.81E-05	0	0	4.60E-05	0.000583
Centipeda	0.000118	0.000227	0	0	4.60E-05	0.000583
Cytophaga	2.98E-05	2.17E-05	0	0	4.60E-05	0.000583
possible_order_07_norank	0.000177	0.000231	0	0	4.60E-05	0.000583
Acetivibrio	6.83E-05	4.51E-05	0	0	4.85E-06	0.000349
Papillibacter	0.002233	0.001367	0.000136	0.000122	5.68E-05	0.000682
Petrimonas	0.00022	0.000132	3.83E-05	2.93E-05	5.68E-05	0.000682
Chryseolinea	0.000159	0.000109	8.08E-07	2.80E-06	6.14E-06	0.000349
Halobacillus	7.88E-05	5.76E-05	5.99E-07	2.08E-06	6.14E-06	0.000349
SubsectionIII_FamilyI_uncultured	5.27E-05	2.96E-05	4.35E-06	5.79E-06	6.23E-05	0.000728
Streptomyces	0.000105	0.000123	8.49E-06	8.59E-06	6.64E-05	0.000755
BD2-2_norank	0.000118	7.97E-05	2.50E-05	1.79E-05	6.99E-05	0.000775
Erysipelotrichaceae_norank	0.002308	0.00111	0.000331	0.000568	8.54E-05	0.000906
Prevotellaceae_uncultured	0.016452	0.012108	0.001686	0.002703	8.57E-05	0.000906
Spirochaeta	0.000111	6.52E-05	3.08E-06	5.78E-06	8.61E-06	0.000349
SB-1_norank	3.92E-05	3.59E-05	2.13E-06	5.38E-06	9.03E-05	0.000907
Saccharofermentans	4.26E-05	3.58E-05	2.18E-06	5.45E-06	9.03E-05	0.000907
Lentzea	1.44E-05	8.53E-06	6.70E-07	2.32E-06	9.33E-05	0.000907

Intestinimonas	5.21E-05	4.68E-05	3.67E-06	7.12E-06	9.40E-05	0.000907
Slackia	0.000411	0.000309	7.11E-06	1.42E-05	9.68E-06	0.000349
Rhodospirillum	0.000319	0.000213	2.22E-05	2.82E-05	9.98E-05	0.000943

Table S3. Bacterial genera distribution in native Tibetans and Han Chinese

Tibetian & Hans	Tibetians only	Hans only
AEGEAN-169_marine_group	12up	Acanthopleuribacter
Acetanaerobacterium	AKYG587	Acaryochloris
Acetatifactor	Abiotrophia	Acetobacter
Acetitomaculum	Actinoplanes	Acetohalobium
Acetivibrio	Actinospica	Acidiferrobacter
Acetobacterium	Actinotalea	Acidiphilium
Acholeplasma	Aeribacillus	Actimicrobium
Achromobacter	Aestuariimicrobium	Actinoalloteichus
Acidaminobacter	Alishewanella	Actinocorallia
Acidaminococcus	Alloiococcus	Advenella
Acidithiobacillus	Alloscardovia	Agaricicola
Acidobacterium	Altererythrobacter	Algibacter
Acidothermus	Aminivibrio	Algicola
Acidovorax	Aminomonas	Aliidiomarina
Acinetobacter	Anaerobacillus	Alkanibacter
Actinobacillus	Anaeroglobus	Alkanindiges
Actinobaculum	Anaerovirgula	Alterococcus
Actinokineospora	Aquabacter	Alysiella
Actinomadura	Aquamicrobium	Anabaena
Actinomyces	Aureispira	Anaeromyxobacter
Adhaeribacter	Azovibrio	Ancylobacter
Adlercreutzia	Bulleidia	Anderseniella
Aequorivita	Buttiauxella	Arcanobacterium
Aeriscardovia	C1-B045	Arhodomonas
Aerococcus	Caldanaerobius	Azohydromonas
Aeromicrobium	Caldibacillus	Azospirillum
Aeromonas	Caldicellulosiruptor	BAL58_marine_group
Afipia	Caldinitratiruptor	Bacteriovorax
Aggregatibacter	Calothrix	Bartonella
Agromyces	Candidatus_Amoebophilus	Bibersteinia
Akkermansia	Candidatus_Blochmannia	Borrelia
Albidiferax	Candidatus_Carsonella	Bowmanella
Alcaligenes	Candidatus_Hepatoplasma	Brachybacterium
Algoriphagus	Candidatus_Planktoluna	Brenneria
Alicyclobacillus	Candidatus_Tammella	Buchnera
Aliivibrio	Cellulosimicrobium	Bythopirellula
Alistipes	Cerasicoccus	Caedibacter
Alkalibacillus	Clostridium_sensu_stricto_18	Caenispirillum
Alkalibacter	Conexibacter	Caldisericum
Alkaliflexus	Coprothermobacter	Calidifontibacter
Alkaliphilus	Coraliomargarita	Caminibacter

Allisonella
Allobaculum
Alloprevotella
Alpinimonas
Alteromonas
Amycolatopsis
Anaerobiospirillum
Anaerobranca
Anaerococcus
Anaerofilum
Anaerofustis
Anaerophaga
Anaeroplasma
Anaerosinus
Anaerosphaera
Anaerospora
Anaerosporobacter
Anaerostipes
Anaerotruncus
Anaerovibrio
Anaerovorax
Aneurinibacillus
Anoxybacillus
Anoxynatronum
Aquabacterium
Aquicella
Aquimarina
Arcicella
Arcobacter
Ardenticatena
Arenibacter
Arenicella
Arenimonas
Arthrobacter
Asteroleplasma
Atopobium
Atopococcus
Atopostipes
Avibacterium
Azoarcus
Azonexus
Azospira
BCf9-17_termite_group
Bacillus
Craurococcus
Crenothrix
Curvibacter
Dehalobacterium
Demequina
Desulfarculus
Desulfatibacillum
Dethiosulfovibrio
Dictyoglomus
Dietzia
Diplosphaera
Dongia
Eggerthia
Elusimicrobium
Enterovibrio
Fabibacter
Frigoribacterium
Geodermatophilus
Geopsychrobacter
Gleocapsa
Haematobacter
Heliobacillus
Hoeflea
Isoptericola
Jannaschia
Kaistia
Kingella
Kitasatospora
Klugiella
Kroppenstedtia
Labrenzia
Lacticigenium
Lampropedia
Lentimonas
Leptobacterium
Magnetococcus
Marixanthomonas
Mesoplasma
Microaerobacter
Microvirgula
Mobiluncus
Moorella
NS2b_marine_group
NS3a_marine_group
Caminicella
Candidatus_Allobeggiatoa
Candidatus_Bacilloplasma
Candidatus_Branchiomonas
Candidatus_Chlorothrix
Candidatus_Flaviluna
Candidatus_Glomeribacter
Candidatus_Gortzia
Candidatus_Hepatincola
Candidatus_Kinetoplastibacterium
Candidatus_Lariskella
Candidatus_Liberibacter
Candidatus_Maribeggiatoa
Candidatus_Marithioploca
Candidatus_Methylacidiphilum
Candidatus_Moranella
Candidatus_Riesia
Candidatus_Scalindua
Candidatus_Tremblaya
Candidatus_Uzinura
Candidatus_Xiphinematobacter
Candidatus_Zinderia
Carboxydocella
Carboxydothermus
Castellaniella
Catenovulum
Catenulispora
Caulobacter
Cedecea
Cellulomonas
Chelatococcus
Chlorobium
Chroococcus
Chrysiogenes
Chthonomonas
Clavibacter
Clostridiisalibacter
Clostridium_sensu_stricto_16
Clostridium_sensu_stricto_9
Collimonas
Costertonia
Croceibacter
Crossiella
Cyclobacterium

Bacteroides	Neptuniibacter	Dehalobium
Balneatrix	Neptunomonas	Delftia
Balneola	Nesterenkonia	Derxia
Barnesiella	OM60(NOR5)_clade	Desulfobacter
Bauldia	Oceanisphaera	Desulfobotulus
Bdellovibrio	Oleibacter	Desulfocella
Bergeyella	Oligella	Desulfococcus
Bhargavaea	Olsenella	Desulfomonile
Bifidobacterium	Ornithobacterium	Desulfonispora
Bilophila	Oscillatoria	Desulforegula
Bisgaardia	Ottowia	Desulforhopalus
Blastocatella	Pelagibius	Desulfotignum
Blastococcus	Phenylobacterium	Desulfurivibrio
Blastopirellula	Photobacterium	Dichotomicrobium
Blautia	Pilimelia	Dyadobacter
Blvii28_wastewater-sludge_group	Pir4_lineage	Emticicia
Bordetella	Plasticicumulans	Enhydrobacter
Brachymonas	Propioniciclava	Ensifer
Brachyspira	Propioniferax	Epilithonimonas
Bradyrhizobium	Pseudaminobacter	Exilispira
Brevibacillus	Pseudoramibacter	Facklamia
Brevibacterium	Puniceicoccus	Ferrovibrio
Brevundimonas	Rhodothalassium	Filimonas
Bryobacter	Roseobacter	Formosa
Burkholderia	Roseobacter_clade_NAC11-7_lineage	Francisella
Butyricicoccus	Roseobacter_clade_OCT_lineage	GAL15
Butyricimonas	Rs-M59_termite_group	GKS98_freshwater_group
Butyrivibrio	Rugamonas	Gaiella
CL500-29_marine_group	SAR92_clade	Gallionella
Caenimonas	Saccharibacter	Gangjinia
Caldanaerobacter	Salinirepens	Geomicrobium
Caldicoprobacter	Sandaracinus	Georgenia
Caldithrix	Schlesneria	Georgfuchsia
Caloramator	Sharpea	Gilvibacter
Campylobacter	Shimazuella	Gracilibacillus
Candidatus_Accumulibacter	Silanimonas	Halarsenatibacter
Candidatus_Actinomarina	Sinomonas	Haliscomenobacter
Candidatus_Alysiosphaera	Skermanella	Halobacteroides
Candidatus_Aquiluna	Sneathia	Haloferula
Candidatus_Armantifilum	Soehngenia	Haloplasma
Candidatus_Arthromitus	Sphaerotilus	Halorhodospira
Candidatus_Cloacamonas	Spirilliplanes	Hansschlegelia

Candidatus_Competibacter	*Sporotomaculum*	*Helicobacter*
Candidatus_Desulforudis	*Streptobacillus*	*Herbiconiux*
Candidatus_Entotheonella	*Thalassobacillus*	*Hirschia*
Candidatus_Koribacter	*Thalassomonas*	*Holophaga*
Candidatus_Phytoplasma	*Thermanaeromonas*	*Hydrogenophilus*
Candidatus_Planktomarina_(DC5-80-3_lineage)	*Thermicanus*	*Hyphomonas*
Candidatus_Planktophila	*Thermoanaerobacterium*	*Ideonella*
Candidatus_Saccharimonas	*Thermodesulfatator*	*Ignavigranum*
Candidatus_Solibacter	*Thermonema*	*Inhella*
Candidatus_Sulcia	*Thermosinus*	*Kerstersia*
Candidatus_Symbiothrix	*Thermotoga*	*Kineococcus*
Candidatus_Thiophysa	*Thioalkalivibrio*	*Kineosporia*
Capnocytophaga	*Uliginosibacterium*	*Kordia*
Carnobacterium	*Viridibacillus*	*Kribbella*
Catenibacterium	*Winogradskyella*	*Ktedonobacter*
Catonella	*ZD0417_marine_group*	*Labrys*
Cellulosilyticum	*marine_group*	*Lactovum*
Cellvibrio	*norank_c__Arctic97B-4_marine_group*	*Larkinella*
Centipeda	*norank_c__BD7-11*	*Leeia*
Cesiribacter	*norank_c__Bacilli*	*Leminorella*
Cetobacterium	*norank_c__DEV055*	*Lentibacter*
Chamaesiphon	*norank_c__Fibrobacteria*	*Leptolinea*
Chitinophaga	*norank_c__ML635J-35*	*Limibacter*
Christensenella	*norank_c__SHA-26*	*Litorilinea*
Chromobacterium	*norank_c__SS1-B-03-39*	*Lonepinella*
Chroococcidiopsis	*norank_c__Spirochaetes*	*Longilinea*
Chryseobacterium	*norank_f__01D2Z36*	*M2PB4-61_termite_group*
Chryseolinea	*norank_f__A0839*	*M2PT2-76_termite_group*
Chryseomicrobium	*norank_f__BVA18*	*Magnetospirillum*
Chthoniobacter	*norank_f__Bacillaceae*	*Magnetovibrio*
Citricoccus	*norank_f__Elev-16S-1166*	*Marine_Methylotrophic_Group_3*
Citrobacter	*norank_f__Elev-16S-1332*	*Marinococcus*
Cloacibacillus	*norank_f__F0723*	*Marinospirillum*
Cloacibacterium	*norank_f__FCPS473*	*Meganema*
Clostridium_sensu_stricto	*norank_f__FFCH16767*	*Meiothermus*
Clostridium_sensu_stricto_1	*norank_f__Family_III*	*Mesoflavibacter*
Clostridium_sensu_stricto_10	*norank_f__Family_XIII*	*Methylobacillus*
Clostridium_sensu_stricto_11	*norank_f__Flavobacteriaceae*	*Methylobacterium*
Clostridium_sensu_stricto_12	*norank_f__HSB_OF53-F07*	*Methylocaldum*
Clostridium_sensu_stricto_13	*norank_f__KCM-B-112*	*Methylococcus*
Clostridium_sensu_stricto_14	*norank_f__KD1-131*	*Methylohalomonas*

Clostridium_sensu_stricto_15	*norank_f__KD3-93*	*Methylophaga*
Clostridium_sensu_stricto_17	*norank_f__Kineosporiaceae*	*Methylorosula*
Clostridium_sensu_stricto_3	*norank_f__Leptotrichiaceae*	*Methyloversatilis*
Clostridium_sensu_stricto_5	*norank_f__Ll142-3M24*	*Methylovorus*
Clostridium_sensu_stricto_7	*norank_f__MSB-1E8*	*Mitsuaria*
Clostridium_sensu_stricto_8	*norank_f__Marine_Methylotrophic_Group_2*	*Mongoliicoccus*
Coleofasciculus	*norank_f__Nitrosomonadaceae*	*Moritella*
Collinsella	*norank_f__OM1_clade*	*Mucispirillum*
Colwellia	*norank_f__P._palm_C_85*	*Mycetocola*
Comamonas	*norank_f__PeH08*	*NS10_marine_group*
Conchiformibius	*norank_f__Plot4-2H12*	*Natronovirga*
Coprobacillus	*norank_f__SBYZ-1017*	*Nevskia*
Coprobacter	*norank_f__SS1-B-06-26*	*Nitrolancea*
Coprococcus	*norank_f__TK85*	*Nitrosomonas*
Coriobacterium	*norank_f__TTA-B15*	*Nitrosospira*
Corynebacterium	*norank_f__TX4CB-01*	*Nitrospina*
Coxiella	*norank_f__Xanthomonadaceae*	*Nocardiopsis*
Crocinitomix	*norank_f__alphaI_cluster*	*Nonomuraea*
Cronobacter	*norank_f__mle1-27*	*Nostoc*
Cryomorpha	*norank_f__possible_family_01*	*Novosphingobium*
Cryptanaerobacter	*norank_o__43F-1404R*	*Oceanobacillus*
Cupriavidus	*norank_o__DSM_5130*	*Ohtaekwangia*
Curtobacterium	*norank_o__Dehalococcoidales*	*Oleiphilus*
Cyanobacterium	*norank_o__E6aD10*	*Orbus*
Cytophaga	*norank_o__Ignavibacteriales*	*Pacificibacter*
Dasania	*norank_o__JFR0501-aaa03c07*	*Paenalcaligenes*
Dechloromonas	*norank_o__JG30-KF-AS9*	*Paracoccus*
Deferrisoma	*norank_o__JG30-KF-CM45*	*Paraeggerthella*
Defluviicoccus	*norank_o__KM16*	*Paramoritella*
Defluviimonas	*norank_o__Lineage_I_(Endomicrobia)*	*Parasegetibacter*
Defluviitalea	*norank_o__MB11C04_marine_group*	*Parasporobacterium*
Deinococcus	*norank_o__R103-B63*	*Parvibaculum*
Dendrosporobacter	*norank_o__RS-B22*	*Parvularcula*
Denitratisoma	*norank_o__Sh765B-AG-111*	*Patulibacter*
Desemzia	*norank_o__Subgroup_11*	*Pedomicrobium*
Desulfatitalea	*norank_o__Subgroup_17*	*Persicirhabdus*

Desulfitibacter
Desulfitobacterium
Desulfobacca
Desulfobulbus
Desulfocapsa
Desulfomicrobium
Desulfonatronum
Desulfonema
Desulfopila
Desulfosporosinus
Desulfotomaculum
Desulfovermiculus
Desulfovibrio
Desulfurispirillum
Desulfurispora
Desulfuromonas
Desulfuromusa
Dethiobacter
Dethiosulfatibacter
Devosia
Dialister
Diaphorobacter
Dickeya
Dokdonella
Dokdonia
Dolosigranulum
Dorea
Dysgonomonas
Edwardsiella
Eggerthella
Ekhidna
Endozoicomonas
Enteractinococcus
Enterobacter
Enterococcus
Enterorhabdus
Epulopiscium

norank_o__Subgroup_5
norank_p__BHI80-139
norank_p__CKC4
norank_p__GOUTA4
norank_p__RsaHF231
uncultured_f__Aerococcaceae
uncultured_f__Anaplasmataceae
uncultured_f__Clostridiaceae_3
uncultured_f__Geodermatophilaceae
uncultured_f__Microbacteriaceae
uncultured_f__Pseudomonadaceae
uncultured_f__Puniceicoccaceae
uncultured_f__Vibrionaceae

Persicitalea
Phaeospirillum
Phaselicystis
Phormidium
Phycisphaera
Pigmentiphaga
Pirellula
Piscinibacter
Planifilum
Plantibacter
Pleurocapsa
Portibacter
Pricia
Procabacter
Propionicimonas
Propionimicrobium
Propionispira
Propionivibrio
Prosthecobacter
Prosthecochloris
Pseudochrobactrum
Pseudoduganella
Pseudorhodoferax
Psychrilyobacter
Psychroflexus
Psychroserpens
Pyramidobacter
Rapidithrix
Rhizobacter
Rhizomicrobium
Rhodoferax
Rhodoplanes
Rhodothermus
Rhodovibrio
Riemerella
Roseiflexus
Roseobacter_clade_CHAB-I-5_lin

Erwinia
Erysipelothrix
Erythrobacter
Escherichia-Shigella
Ethanoligenens
Eubacterium
Euhalothece
Euzebya
Ewingella
Exiguobacterium
Faecalibacterium
Fastidiosipila
Ferrithrix
Ferritrophicum
Ferruginibacter
Fibrella
Fibrobacter
Fictibacillus
Filifactor
Filomicrobium
Finegoldia
Flavisolibacter
Flavobacterium
Flavonifractor
Flectobacillus
Flexibacter
Fluviicola
Fonticella
Fusibacter
Fusobacterium
Gaetbulibacter
Gallibacterium
Garciella
Gardnerella
Gelria
Gemella
Gemmata
Gemmatimonas
Gemmobacter
Geobacillus
Geobacter
Gilliamella
Gillisia

eage
Roseomonas
Roseovarius
Rubritalea
Rudanella
Salinicoccus
Salinimicrobium
Salinisphaera
Saprospira
Schlegelella
Scytonema
Sebaldella
Sediminibacterium
Shinella
Simiduia
Sinobaca
Sinomicrobium
Snodgrassella
Snowella
Sphingopyxis
Sphingorhabdus
Spirulina
Sporolactobacillus
Sterolibacterium
Sulfurihydrogenibium
Swaminathania
Symploca
Syntrophobotulus
Tabrizicola
Telmatobacter
Telmatospirillum
Tepidimonas
Tepidiphilus
Termite_Treponema_cluster
Tetragenococcus
Thalassobius
Thermoanaerobaculum
Thermobacillus
Thermodesulforhabdus
Thermolithobacter
Thermosporothrix
Thioalkalispira
Thiomonas
Tistlia

Glaciecola
Globicatella
Gloeocalita
Gordonibacter
Gracilibacter
Gramella
Granulicatella
Granulicella
Granulosicoccus
Guggenheimella
Haemophilus
Hafnia
Halanaerobium
Haliangium
Haliea
Halobacillus
Halochromatium
Halolactibacillus
Halomonas
Halothiobacillus
Helcococcus
Heliobacterium
Herbaspirillum
Herminiimonas
Hespellia
Holdemania
Howardella
Hydrogenoanaerobacterium
Hydrogenophaga
Hymenobacter
Hyphomicrobium
Iamia
Idiomarina
Ignatzschineria
Ignavibacterium
Ilumatobacter
Incertae_Sedis
Inquilinus
Intestinimonas
Isosphaera
Janthinobacterium
Jeotgalibacillus
Jeotgalicoccus
Tropicimonas
Vitreoscilla
Vogesella
Wautersiella
Weeksella
Xanthobacter
Xenophilus
Zeaxanthinibacter
Zobellia
Zoogloea
norank_c__ARKDMS-49
norank_c__Gammaproteobacteria
norank_c__JG30-KF-CM66
norank_c__JG37-AG-4
norank_c__KZNMV-5-B42
norank_c__LD1-PB3
norank_c__ML602M-17
norank_c__MSB-5B2
norank_c__Opitutae
norank_c__Phycisphaerae
norank_c__SGST604
norank_c__WCHB1-32
norank_c__WCHB1-41
norank_f__08D2Z94_hypersaline_microbial_mat_group
norank_f__09D2Z46
norank_f__09D2Z48
norank_f__1174-901-12
norank_f__1921-2
norank_f__288-2
norank_f__AKIW1012
norank_f__AKYH767
norank_f__AT-s3-44
norank_f__B01R012
norank_f__B2706-C7
norank_f__BPS-CK174
norank_f__BSV40
norank_f__BacC-u-018
norank_f__CM1G08
norank_f__CWT_CU03-E12
norank_f__Christensenellaceae
norank_f__Cryptosporangiaceae
norank_f__Cystobacteraceae
norank_f__DA101_soil_group

Johnsonella
Klebsiella
Kluyvera
Kocuria
Krasilnikovia
Kurthia
Lachnoanaerobaculum
Lachnospira
Lacibacter
Lactobacillus
Lactococcus
Lautropia
Lawsonia
Leadbetterella
Legionella
Leifsonia
Lentibacillus
Lentisphaera
Lentzea
Leptolyngbya
Leptospira
Leptothrix
Leptotrichia
Leucobacter
Leuconostoc
Lewinella
Limnobacter
Limnohabitans
Listeria
Loktanella
Luteibacter
Luteimonas
Lutibacter
Lutimonas
Lutispora
Lysinibacillus
Lysinimonas
Lysobacter
MWH-UniP1_aquatic_group
Macellibacteroides
Macrococcus

norank_f__DUNssu371
norank_f__Deep_1
norank_f__EF100-94H03
norank_f__FamilyI
norank_f__GOUTB8
norank_f__GoM-GC232-4463-Bac1
norank_f__JTB215
norank_f__KF-JG30-B3
norank_f__LD12_freshwater_group
norank_f__ME2
norank_f__MWH-CFBk5
norank_f__Milano-WF1B-03
norank_f__NS7_marine_group
norank_f__Nannocystaceae
norank_f__Oligosphaeraceae
norank_f__PAUC26f
norank_f__Paenibacillaceae
norank_f__PeH15
norank_f__Peptostreptococcaceae
norank_f__Porphyromonadaceae
norank_f__Promicromonosporaceae
norank_f__R103-B20
norank_f__S15A-MN91
norank_f__S25-593
norank_f__SHWN-night2
norank_f__SR-FBR-L83
norank_f__Sva1033
norank_f__Z4MB62
norank_f__cvE6
norank_f__gir-aah93h0
norank_f__s74e-6049
norank_f__type_III
norank_o__Armatimonadales
norank_o__B1-7BS
norank_o__BC-COM435
norank_o__Bacteroidales
norank_o__C-2
norank_o__Cytophagales
norank_o__DTB120
norank_o__FW113
norank_o__GIF9

Mahella	*norank_o__GZKB75*
Mangroviflexus	*norank_o__Halanaerobiales*
Mannheimia	*norank_o__KD3-62*
Maribacter	*norank_o__Lineage_IIc*
Marinicella	*norank_o__ML-A-10*
Marinifilum	*norank_o__MSBL5*
Mariniradius	*norank_o__NRB23*
Marinobacter	*norank_o__SAR11_clade*
Marinobacterium	*norank_o__SS1-B-09-64*
Marinoscillum	*norank_o__Solirubrobacterales*
Mariprofundus	*norank_o__Subgroup_9*
Maritimibacter	*norank_o__TPD-58*
Maritimimonas	*norank_o__WD2101_soil_group*
Marmoricola	*norank_o__d142*
Marvinbryantia	*norank_p__Candidate_division_OP9*
Massilia	*norank_p__GAL08*
Megamonas	*norank_p__Hyd24-12*
Megasphaera	*norank_p__aquifer1*
Melioribacter	*possible_genus_05*
Meniscus	*pt46*
Mesorhizobium	*uncultured_f__Acetobacteraceae*
Methylibium	*uncultured_f__Chlamydiaceae*
Methylophilus	*uncultured_f__Deferribacteraceae*
Methylotenera	*uncultured_f__Gracilibacteraceae*
Microbacterium	*uncultured_f__Halobacteroidaceae*
Micrococcus	*uncultured_f__Legionellaceae*
Microcystis	*uncultured_f__Micrococcaceae*
Microlunatus	*uncultured_f__Moraxellaceae*
Micromonospora	*uncultured_f__Rickettsiaceae*
Microvirga	*uncultured_f__Simkaniaceae*
Mitsuokella	*uncultured_f__Sphingomonadaceae*
Modestobacter	*uncultured_f__Sporichthyaceae*
Mogibacterium	*uncultured_f__Thermoactinomycetaceae*
Moraxella	*uncultured_o__Bacillales*
Morganella	*uncultured_o__Chloroflexales*
Moryella	*uncultured_o__Rhizobiales*
Mucilaginibacter	*uncultured_o__Rickettsiales*
Murdochiella	*uncultured_o__Sphingomonadales*
Muricauda	
Mycobacterium	

Mycoplasma
Myroides
NS4_marine_group
NS5_marine_group
Nafulsella
Nannocystis
Negativicoccus
Neisseria
Niabella
Nitratifractor
Nitratireductor
Nitriliruptor
Nitritalea
Nitrobacter
Nitrosococcus
Nitrospira
Nocardia
Nocardioides
Nonlabens
Nordella
Nosocomiicoccus
Noviherbaspirillum
OM27_clade
OM43_clade
Oceanicaulis
Ochrobactrum
Odoribacter
Oligosphaera
Olivibacter
Opitutus
Oribacterium
Ornatilinea
Oryzihumus
Oscillibacter
Oscillospira
Otariodibacter
Owenweeksia
Oxalobacter
Oxobacter
PRD01a011B
Paenibacillus
Paludibacter
Pantoea
Papillibacter

Parabacteroides
Parapedobacter
Paraprevotella
Parasutterella
Parvibacter
Parvimonas
Pasteurella
Paucimonas
Paucisalibacillus
Pectinatus
Pectobacterium
Pediococcus
Pedobacter
Pelobacter
Pelomonas
Pelosinus
Pelospora
Pelotomaculum
Peptococcus
Peptoniphilus
Peptostreptococcus
Peredibacter
Petrimonas
Phascolarctobacterium
Phocaeicola
Phocoenobacter
Phycicoccus
Phyllobacterium
Planctomyces
Planococcus
Planomicrobium
Plesiomonas
Polaribacter
Polaromonas
Polynucleobacter
Pontibacillus
Pontibacter
Porphyromonas
Prevotella
Prochlorococcus
Prochlorothrix
Prolixibacter
Promicromonospora
Propionibacterium

Propionicicella
Propionispora
Proteiniborus
Proteiniclasticum
Proteiniphilum
Proteocatella
Proteus
Providencia
Pseudoalteromonas
Pseudobutyrivibrio
Pseudoclavibacter
Pseudoflavonifractor
Pseudofulvimonas
Pseudolabrys
Pseudomonas
Pseudonocardia
Pseudospirillum
Pseudoxanthomonas
Psychrobacter
Psychromonas
Pusillimonas
Quadrisphaera
Quinella
RC9_gut_group
Rahnella
Ralstonia
Ramlibacter
Raoultella
Reichenbachiella
Reyranella
Rheinheimera
Rhizobium
Rhodanobacter
Rhodobacter
Rhodobium
Rhodococcus
Rhodocytophaga
Rhodoglobus
Rhodopirellula
Rhodopseudomonas
Rhodospirillum
Rhodovulum
Rickettsia
Rikenella

Robinsoniella
Roseateles
Roseburia
Roseibacillus
Rothia
Rubellimicrobium
Rubrobacter
Ruegeria
Ruminobacter
Ruminococcus
SEEP-SRB1
SEEP-SRB4
SM1A02
SP3-e08
Saccharofermentans
Saccharopolyspora
Salegentibacter
Salinibacter
Salmonella
Sarcina
Scardovia
Schleiferia
Sedimentibacter
Sediminibacter
Sediminimonas
Segetibacter
Selenomonas
Serratia
Shewanella
Shimwellia
Shuttleworthia
Sideroxydans
Simplicispira
Singulisphaera
Siphonobacter
Slackia
Smithella
Sodalis
Solibacillus
Solirubrobacter
Solitalea
Solobacterium
Sorangium
Sphingobacterium

Sphingobium
Sphingomonas
Sphingosinicella
Spirochaeta
Spiroplasma
Spirosoma
Sporobacter
Sporomusa
Sporosarcina
Staphylococcus
Stenotrophomonas
Steroidobacter
Stomatobaculum
Streptococcus
Streptomyces
Subdoligranulum
Succinatimonas
Succiniclasticum
Succinimonas
Succinispira
Succinivibrio
Sufflavibacter
Sulfitobacter
Sulfobacillus
Sulfuricella
Sulfuricurvum
Sulfurimonas
Sulfuritalea
Sulfurospirillum
Sulfurovum
Sunxiuqinia
Sutterella
Sva0081_sediment_group
Symbiobacterium
Synechococcus
Synechocystis
Syntrophococcus
Syntrophomonas
Syntrophorhabdus
Syntrophus
Tannerella
Tatumella
Tenacibaculum
Tepidimicrobium

Terrimonas
Tessaracoccus
Tetrasphaera
Thalassospira
Thauera
Thermacetogenium
Thermanaerovibrio
Thermincola
Thermobifida
Thermomonas
Thermovirga
Thiobacillus
Thiohalocapsa
Thiohalorhabdus
Thiomicrospira
Thiothrix
Tissierella
Tolumonas
Treponema
Trichococcus
Tropicibacter
Truepera
Tumebacillus
Tunicatimonas
Turicibacter
Turneriella
U29-B03
Ulvibacter
Undibacterium
Ureaplasma
Ureibacillus
Vagococcus
Vallitalea
Varibaculum
Variovorax
Veillonella
Verrucomicrobium
Vibrio
Victivallis
Virgibacillus
Weissella
Xanthomonas
Xenorhabdus
Yersinia

Zymomonas
dgA-11_gut_group
endosymbionts
hgcI_clade
hoa5-07d05_gut_group
norank_c__055B07-P-DI-P58
norank_c__AEGEAN-245
norank_c__Acidobacteria
norank_c__Actinobacteria
norank_c__BD2-2
norank_c__BSV13
norank_c__Cyanobacteria
norank_c__Gitt-GS-136
norank_c__OM190
norank_c__OPB35_soil_group
norank_c__OPB54
norank_c__Oligosphaeria
norank_c__Pla3_lineage
norank_c__Pla4_lineage
norank_c__R76-B128
norank_c__RFP12_gut_group
norank_c__S085
norank_c__SAR202_clade
norank_c__SB-1
norank_c__SB-5
norank_c__SC3-20
norank_c__SM1A07
norank_c__TA18
norank_c__TK10
norank_c__VC2.1_Bac22
norank_c__vadinHA17
norank_c__vadinHA49
norank_f__0319-6G20
norank_f__0319-6M6
norank_f__21f08
norank_f__480-2
norank_f__64K2
norank_f__9M32
norank_f__ABS-19
norank_f__Actinomycetaceae
norank_f__Alcanivoracaceae
norank_f__BS11_gut_group
norank_f__BSV26
norank_f__CA002

norank_f__CAP-aah99b04
norank_f__CFT112H7
norank_f__CMW-169
norank_f__CR-115
norank_f__Chlorobiaceae
norank_f__Comamonadaceae
norank_f__Coriobacteriaceae
norank_f__DA111
norank_f__DEV007
norank_f__DUNssu044
norank_f__Defluviitaleaceae
norank_f__E6aC02
norank_f__Enterobacteriaceae
norank_f__Erysipelotrichaceae
norank_f__FTLpost3
norank_f__GR-WP33-58
norank_f__Hados.Sed.Eubac.3
norank_f__Hyphomonadaceae
norank_f__I-10
norank_f__IheB3-7
norank_f__JTB255_marine_benthic_group
norank_f__LCP-89
norank_f__LD29
norank_f__LH041
norank_f__LWSR-14
norank_f__Lachnospiraceae
norank_f__Ll142-1A4
norank_f__M2PB4-65_termite_group
norank_f__MA-28-I98C
norank_f__MAT-CR-H4-C10
norank_f__MAT-CR-H6-H10
norank_f__MAT-CR-P4-C12
norank_f__MBAE14
norank_f__ML635J-40_aquatic_group
norank_f__Marinilabiaceae
norank_f__Methylophilaceae
norank_f__MgMjR-022
norank_f__NS11-12_marine_group
norank_f__NS9_marine_group
norank_f__OM182_clade

norank_f__OPB56
norank_f__Oxalobacteraceae
norank_f__P._palm_C-A_51
norank_f__P5D1-392
norank_f__PAUC43f_marine_bent hic_group
norank_f__PHOS-HE36
norank_f__PHOS-HE51
norank_f__PL-11B10
norank_f__PL-11B8_wastewater-sl udge_group
norank_f__Pasteurellaceae
norank_f__Prevotellaceae
norank_f__RB41
norank_f__RF16
norank_f__RH-aaj90h05
norank_f__Rhodocyclaceae
norank_f__Rs-E47_termite_group
norank_f__Ruminococcaceae
norank_f__S0134_terrestrial_grou p
norank_f__S24-7
norank_f__SAR116_clade
norank_f__SAR406_clade(Marine_ group_A)
norank_f__SAR86_clade
norank_f__SBYC
norank_f__SJA-149
norank_f__SM2D12
norank_f__SRB2
norank_f__ST-12K33
norank_f__Sandaracinaceae
norank_f__Sphingobacteriaceae
norank_f__Sporichthyaceae
norank_f__Surface_1
norank_f__Surface_2
norank_f__Surface_4
norank_f__Sva0725
norank_f__Sva0996_marine_group
norank_f__TSAC18
norank_f__Veillonellaceae
norank_f__WCHB1-69
norank_f__ZD0405
norank_f__boneC3G7

norank_f__env.OPS_17
norank_f__livecontrolB21
norank_f__mitochondria
norank_f__p-2534-18B5_gut_grou
p
norank_f__possible_family_02
norank_f__ratAN060301C
norank_f__vadinBB60
norank_o__34P16
norank_o__AK4AB2-03B
norank_o__AKIW781
norank_o__AT425-EubC11_terrest
rial_group
norank_o__Adriatic90
norank_o__Ardenticatenales
norank_o__B38
norank_o__BD2-11_terrestrial_gr
oup
norank_o__BD7-8_marine_group
norank_o__BD72BR169
norank_o__C0119
norank_o__CCM11a
norank_o__Caenarcaniphilales
norank_o__Chthonomonadales
norank_o__D8A-2
norank_o__DB1-14
norank_o__E01-9C-26_marine_gr
oup
norank_o__EC3
norank_o__FGL12-B44
norank_o__GR-WP33-30
norank_o__Gastranaerophilales
norank_o__HOC36
norank_o__KI89A_clade
norank_o__Kazan-3B-09
norank_o__Lineage_IIa
norank_o__Lineage_IIb
norank_o__Lineage_IV
norank_o__NB1-n
norank_o__NKB5
norank_o__OCS116_clade
norank_o__Obscuribacterales
norank_o__Oligosphaerales
norank_o__P131-4

norank_o__PeM15
norank_o__Pla1_lineage
norank_o__RF9
norank_o__RS-F29
norank_o__Run-SP154
norank_o__SAR324_clade(Marine _group_B)
norank_o__SB1-18
norank_o__SC-I-84
norank_o__Sh765B-TzT-29
norank_o__Subgroup_18
norank_o__Subgroup_2
norank_o__Subgroup_21
norank_o__Subgroup_23
norank_o__Subgroup_25
norank_o__Subgroup_4
norank_o__Subgroup_6
norank_o__Subgroup_7
norank_o__Sva0071
norank_o__Sva0485
norank_o__TRA3-20
norank_o__UCT_N117
norank_o__Vampirovibrionales
norank_o__aaa34a10
norank_o__possible_order_07
norank_o__vadinBA26
norank_o__vadinHA64
norank_p__Armatimonadetes
norank_p__BD1-5
norank_p__Candidate_division_B RC1
norank_p__Candidate_division_JS 1
norank_p__Candidate_division_K B1
norank_p__Candidate_division_O D1
norank_p__Candidate_division_O P11
norank_p__Candidate_division_O P3
norank_p__Candidate_division_O P8
norank_p__Candidate_division_SR

1
norank_p__Candidate_division_T
M7
norank_p__Candidate_division_W
S3
norank_p__Candidate_division_W
S6
norank_p__JL-ETNP-Z39
norank_p__NPL-UPA2
norank_p__S2R-29
norank_p__SBYG-2791
norank_p__SHA-109
norank_p__SM2F11
norank_p__TA06
norank_p__TM6
norank_p__WCHB1-60
possible_genus_03
possible_genus_04
possible_genus_Sk003-Sk004
probable_genus_10
uncultured_c__Ardenticatenia
uncultured_c__Cyanobacteria
uncultured_f__Acidaminococcacea
e
uncultured_f__Acidimicrobiaceae
uncultured_f__Acidobacteriaceae_(Subgroup_1)
uncultured_f__Alcaligenaceae
uncultured_f__Alteromonadaceae
uncultured_f__Anaerolineaceae
uncultured_f__Bacillaceae
uncultured_f__Bacteriovoracaceae
uncultured_f__Beijerinckiaceae
uncultured_f__Bradyrhizobiaceae
uncultured_f__Caldilineaceae
uncultured_f__Cardiobacteriaceae
uncultured_f__Carnobacteriaceae
uncultured_f__Caulobacteraceae
uncultured_f__Chitinophagaceae
uncultured_f__Christensenellaceae
uncultured_f__Chromatiaceae
uncultured_f__Clostridiaceae_1
uncultured_f__Clostridiaceae_4
uncultured_f__Comamonadaceae
uncultured_f__Coriobacteriaceae

uncultured_f__Corynebacteriaceae
uncultured_f__Coxiellaceae
uncultured_f__Cryomorphaceae
uncultured_f__Cyclobacteriaceae
uncultured_f__Cytophagaceae
uncultured_f__Defluviitaleaceae
uncultured_f__Dermatophilaceae
uncultured_f__Desulfarculaceae
uncultured_f__Desulfobacteraceae
uncultured_f__Desulfobulbaceae
uncultured_f__Desulfohalobiaceae
uncultured_f__Desulfovibrionaceae
uncultured_f__Desulfurellaceae
uncultured_f__Enterobacteriaceae
uncultured_f__Erysipelotrichaceae
uncultured_f__Erythrobacteraceae
uncultured_f__Eubacteriaceae
uncultured_f__FamilyI
uncultured_f__Family_XI
uncultured_f__Family_XII
uncultured_f__Family_XIII
uncultured_f__Family_XVII
uncultured_f__Family_XVIII
uncultured_f__Fibrobacteraceae
uncultured_f__Flammeovirgaceae
uncultured_f__Flavobacteriaceae
uncultured_f__Gallionellaceae
uncultured_f__Gemmatimonadaceae
uncultured_f__Halothiobacillaceae
uncultured_f__Helicobacteraceae
uncultured_f__Heliobacteriaceae
uncultured_f__Holosporaceae
uncultured_f__Hydrogenophilaceae
uncultured_f__Hyphomicrobiaceae
uncultured_f__Hyphomonadaceae
uncultured_f__Ktedonobacteraceae
uncultured_f__Lachnospiraceae
uncultured_f__Leptospiraceae
uncultured_f__Leptotrichiaceae
uncultured_f__Marinilabiaceae

uncultured_f__Methylophilaceae
uncultured_f__Mycoplasmataceae
uncultured_f__Neisseriaceae
uncultured_f__Nitrosomonadaceae
uncultured_f__Nitrospinaceae
uncultured_f__Oxalobacteraceae
uncultured_f__Paenibacillaceae
uncultured_f__Pasteurellaceae
uncultured_f__Peptococcaceae
uncultured_f__Peptostreptococcaceae
uncultured_f__Phyllobacteriaceae
uncultured_f__Piscirickettsiaceae
uncultured_f__Planctomycetaceae
uncultured_f__Planococcaceae
uncultured_f__Porphyromonadaceae
uncultured_f__Prevotellaceae
uncultured_f__Rhodobacteraceae
uncultured_f__Rhodocyclaceae
uncultured_f__Rhodospirillaceae
uncultured_f__Rhodothermaceae
uncultured_f__Rikenellaceae
uncultured_f__Ruminococcaceae
uncultured_f__Saprospiraceae
uncultured_f__Solimonadaceae
uncultured_f__Sphingobacteriaceae
uncultured_f__Spirochaetaceae
uncultured_f__Succinivibrionaceae
uncultured_f__Synergistaceae
uncultured_f__Syntrophaceae
uncultured_f__Syntrophobacteraceae
uncultured_f__Syntrophomonadaceae
uncultured_f__Thermoanaerobacteraceae
uncultured_f__Thiotrichaceae
uncultured_f__Veillonellaceae
uncultured_f__Verrucomicrobiaceae
uncultured_f__Xanthobacteraceae
uncultured_f__Xanthomonadaceae

uncultured_f__Xanthomonadales_Incertae_Sedis
uncultured_o__Acidimicrobiales
uncultured_o__Bacteroidales
uncultured_o__Clostridiales
uncultured_o__Corynebacteriales
uncultured_o__Frankiales
uncultured_o__Gaiellales
uncultured_o__Myxococcales
uncultured_o__Order_III
uncultured_o__Selenomonadales
uncultured_o__SubsectionII
uncultured_o__Xanthomonadales
uncultured_p__Chloroflexi
vadinBC27_wastewater-sludge_group

Table S4A. Antibiotic resistant genes identified in gut microbiota from the Han samples

Sample ID / Gene type	0	1	2	3	4	5	6	7	9	11	12	13
**aac6ib**	1470	0	0	0	0	0	0	0	0	0	0	0
**aac6ie**	0	2	14	22	142	0	0	2	0	0	112	4
**acra**	7322	0	22	2	2	12	4	0	36	30	0	2
**acrb**	45758	6	72	2	16	86	2	6	118	130	4	6
**ant3ia**	0	0	0	114	0	2	4	0	0	0	0	122
**ant6ia**	0	66	70	28	72	42	10	96	68	30	48	154
**aph33ib**	12	0	152	8	450	4	4	0	2	0	88	62
**aph3ia**	0	0	76	0	0	0	0	0	0	0	2	0
**aph3iiia**	6	12	16	10	16	90	80	4	0	4	4	38
**aph6id**	24	0	230	4	554	6	8	0	0	0	106	90
**arna**	14630	0	26	0	4	18	0	0	28	34	0	0
**baca**	7640	7046	7728	6924	7376	6574	5782	7192	8312	6758	4458	8152
**bcr**	8806	0	10	2	0	6	0	0	14	6	0	4
**bl1_ec**	10204	0	16	0	2	2	0	0	24	32	0	2
**bl2d_oxa1**	5898	0	0	0	0	0	0	0	2	0	0	0
**bl2e_cbla**	0	12	188	74	118	80	4	138	34	18	144	22
**bl2e_cepa**	0	46	152	12	158	268	42	36	176	88	152	90
**bl2e_cfxa**	0	1152	704	6	2854	396	706	1438	0	0	284	1046
**bl3_ccra**	78	0	0	0	0	122	138	0	0	0	34	0
**emrd**	5400	4	8	0	4	6	0	0	12	10	0	2
	1596	0	0	0	2	0	0	0	8	14	0	0
**ermb**	8	1168	716	248	476	732	374	116	172	182	832	966
**ermf**	4	162	127	305	2076	144	948	281	1154	956	1718	136

			6	4		4		2				0
**ermg**	0	5016	2	2	0	2	0	0	0	0	14	0
**ermx**	2	22	142	92	36	52	42	52	58	16	22	112
**ksga**	7204	4	26	0	4	16	0	0	26	34	0	0
**macb**	14492	0	16	0	6	32	0	0	20	46	0	8
**mdfa**	7624	2	2	0	0	6	4	0	18	28	0	0
**mdte**	9140	0	16	0	4	18	0	4	14	32	4	0
**mdtf**	24656	4	42	0	16	44	2	0	68	50	0	0
**mdtg**	8162	0	8	0	2	8	4	0	20	26	0	2
**mdth**	7612	0	14	0	4	20	0	2	18	18	0	0
**mdtk**	8702	0	16	0	2	4	2	2	8	28	0	0
**mdtl**	8246	2	10	2	0	12	6	2	12	8	0	0
**mdtm**	12672	0	22	0	0	16	0	2	16	16	0	2
**mdtn**	7526	0	6	0	6	18	0	0	4	16	0	0
**mdto**	13838	0	28	0	4	22	6	0	28	54	0	0
**mdtp**	13010	6	24	2	6	6	0	0	26	34	0	4
**mefa**	0	4	0	0	238	4	0	0	0	2	8	0
**rosa**	1486	0	6	0	0	0	0	0	2	4	0	0
**rosb**	6896	0	20	0	2	10	0	0	10	16	2	0
**sul1**	11848	0	2	180	0	12	0	0	6	4	0	138
**sul2**	18	0	424	0	176	2	0	294	0	0	144	124
**tet32**	2	196	418	8	362	250	114	18	464	388	336	542
**tet37**	0	6914	4	0	2	0	2	2	0	0	0	0
**tet40**	64	454	147	360	1666	570	135	187	1154	1194	906	538
			2				2	2				0
**teta**	68	2	16	2	618	12	0	2	12	38	122	2
**tetb**	62	0	0	4	0	0	0	0	0	0	0	0
**tetc**	5432	0	0	300	0	2	4	0	0	0	0	178
**tetm**	4	2	10	0	0	212	50	0	0	0	18	2

**teto**	30	850	542	112	1580	544	1314	548	542	606	1322	1986
**tetq**	144	17388	7722	26300	24760	11988	6930	22124	12898	10490	8314	7146
**tetw**	0	104	714	128	904	240	74	558	346	286	286	432
**tetx**	0	0	0	330	62	48	0	0	0	0	810	950
**tolc**	11602	2	10	0	4	20	0	2	30	38	0	0
**vanra**	0	0	0	0	0	0	0	0	0	0	0	0
**vanug**	0	8	20	2	4	2	2	14	6	8	4	0
**vanwg**	0	0	0	0	0	0	0	0	0	0	0	18

Table S4B. Antibiotic resistant genes identified in gut microbiota from the Tibetan samples

Sample ID / Gene type	**3_1**	**3_2**	**3_3**	**3_4**	**3_5**	**3_6**	**3_7**	**3_8**	**3_9**	**3_10**	**3_11**	**3_12**	**3_13**	**3_14**	**3_15**
**aac6ie**	50	136	372	500	142	378	1442	828	582	246	3302	28	3978	8	336
**acrb**	46	0	4	82	4	12	0	4926	12	0	378	2	58	2448	230
**ant6ia**	294	200	510	484	208	324	458	182	40	300	546	320	1180	6	254
**aph33ib**	10	0	52	16	12	34	36	2	0	0	74	8	2	822	16
**aph3iiia**	0	0	0	0	4	2	0	14	0	0	4	0	42	0	0
**baca**	3268	2716	3506	4574	3832	3306	2042	2720	2134	3640	4350	2600	4646	2008	3958
**bcr**	0	0	0	12	0	2	0	756	0	0	40	0	4	98	26
**bl2be_ctxm**	0	0	0	0	0	0	0	0	0	0	2	0	0	0	46
**bl2e_cepa**	4	2	0	4	26	6	0	0	0	16	66	0	8	0	2
**bl2e_cfxa**	4874	10578	13150	110	5552	9498	24628	9012	25938	6362	4120	9154	4602	562	15582
**bl3_ccra**	0	0	0	2	0	0	0	0	0	0	6	4	12	0	16
**cata11**	2	0	0	0	0	18	0	4	0	0	14	0	0	0	16
**dfra17**	10	0	0	0	0	0	0	0	0	0	44	0	0	0	6
**emrd**	18	0	2	12	0	0	0	330	0	0	52	0	22	670	62
**emre**	2	0	0	10	0	0	0	0	0	0	24	0	6	4	6
**erma**	0	0	0	0	0	0	0	0	0	0	26	0	2	0	0
**ermg**	0	0	36	0	0	2	0	0	0	1568	0	4	18	0	16
**macb**	16	0	0	14	2	8	0	1234	2	0	60	0	26	606	56
**mdfa**	6	0	0	10	0	10	0	714	0	0	62	2	28	168	46

**mdtf**	18	0	0	32	4	6	0	2600	8	0	134	0	22	1048	118
**mdtg**	10	0	0	12	2	2	0	872	0	0	68	0	18	480	48
**mdth**	8	0	0	2	0	4	0	694	0	2	60	0	12	334	50
**mdtk**	4	0	0	4	0	4	2	764	0	0	34	0	6	244	42
**mdtl**	6	0	0	6	0	2	0	0	0	0	18	0	8	224	6
**mdtm**	14	2	0	12	2	10	2	1340	0	0	70	0	24	230	46
**mdto**	8	0	0	6	2	6	0	1576	0	2	78	0	18	528	46
**mefa**	0	0	0	26	0	0	0	0	0	38	0	6	0	0	0
**mexy**	0	0	2	12	0	0	0	0	0	14	0	2	0	0	0
**rosb**	16	0	0	12	0	2	0	1082	4	0	70	0	16	718	46
**sul2**	14	2	68	40	8	78	124	226	2	0	178	12	20	26	34
**tet**	0	0	2	0	0	0	0	0	0	0	0	0	0	0	2
**tet32**	490	380	418	1078	762	596	454	108	278	384	410	810	1164	40	1064
**tet37**	238	150	310	188	544	300	1354	94	3516	426	0	362	120	2286	562
**tet40**	2594	1274	1964	2430	2496	1336	1044	508	830	1068	840	952	2324	1926	2944
**tetb**	0	0	0	0	0	0	0	0	0	0	0	0	0	10	0
**tetc**	26	0	0	2	0	0	0	0	0	0	84	6	0	0	2
**tetm**	424	92	262	138	518	130	146	108	34	142	924	212	610	0	308
**teto**	1794	770	1544	862	1076	1142	538	484	626	1426	1194	612	2084	38	2952
**tetpa**	2	14	2	8	0	28	8	6	16	0	0	4	2	0	12
**tetpb**	18	10	14	4	0	128	6	8	56	2	4	8	10	0	14

**tetq**	6396	7458	6754	6484	8664	6564	16626	6014	50430	2014	9292	13000	6418	606	11194
**tetw**	2964	1874	3094	5376	2682	1628	1750	236	942	1742	3010	2378	5022	1156	3478
**vang**	12	4	16	492	0	10	6	0	10	28	0	134	10	0	20
**vanra**	0	0	12	16	6	0	0	0	0	0	0	0	0	0	0
**vanrd**	22	0	8	8	6	0	26	0	0	6	8	0	12	2	2
**vanrg**	28	18	44	252	22	14	70	134	46	112	16	42	20	4	48
**vantg**	2	18	44	886	8	14	6	0	18	46	0	182	26	0	40
**vanug**	62	16	104	238	78	56	48	30	2	156	130	52	26	6	112
**vanxyg**	2	0	2	138	2	0	0	0	2	2	0	14	2	0	8
**vatb**	4	0	0	18	0	0	0	0	0	0	0	14	0	0	0

Figure S1

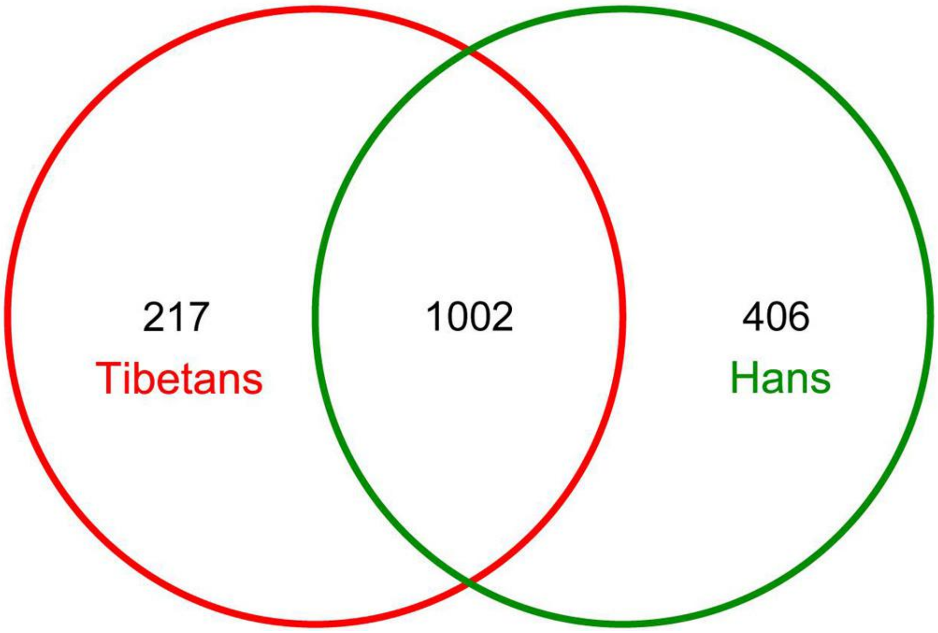

Figure S2

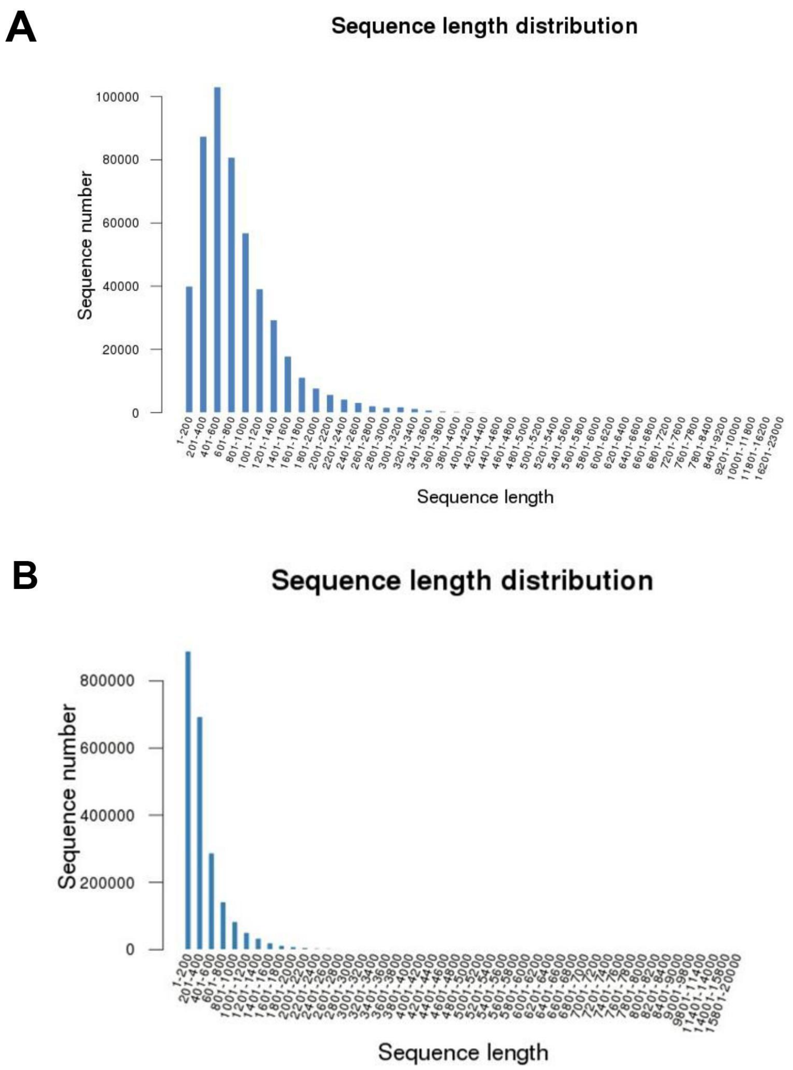

A
Sequence length distribution
Sequence number
100000
80000
60000
40000
20000
0
Sequence length
B
Sequence length distribution
Sequence number
800000
600000
400000
200000
0
Sequence length

Figure S3

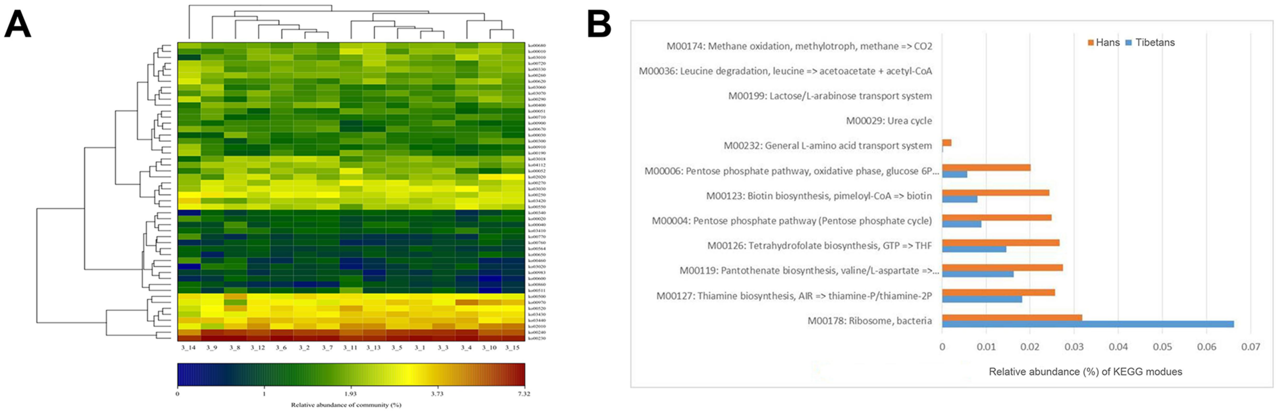

A
3_14
3_9
3_8
3_12
3_6
3_2
3_7
3_11
3_13
3_5
3_1
3_3
3_4
3_10
3_15
0
1
1.93
3.73
7.32
Relative abundance of community (%)
B
Hans
Tibetans
M00174: Methane oxidation, methylotroph, methane => CO2
M00036: Leucine degradation, leucine => acetoacetate + acetyl-CoA
M00199: Lactose/L-arabinose transport system
M00029: Urea cycle
M00232: General L-amino acid transport system
M00006: Pentose phosphate pathway, oxidative phase, glucose 6P...
M00123: Biotin biosynthesis, pimeloyl-CoA => biotin
M00004: Pentose phosphate pathway (Pentose phosphate cycle)
M00126: Tetrahydrofolate biosynthesis, GTP => THF
M00119: Pantothenate biosynthesis, valine/L-aspartate =>...
M00127: Thiamine biosynthesis, AIR => thiamine-P/thiamine-2P
M00178: Ribosome, bacteria
0
0.01
0.02
0.03
0.04
0.05
0.06
0.07
Relative abundance (%) of KEGG modues
